# The genome of the extremophile *Artemia* provides insight into strategies to cope with extreme environments

**DOI:** 10.1186/s12864-021-07937-z

**Published:** 2021-08-31

**Authors:** Stephanie De Vos, Stephane Rombauts, Louis Coussement, Wannes Dermauw, Marnik Vuylsteke, Patrick Sorgeloos, James S. Clegg, Ziro Nambu, Filip Van Nieuwerburgh, Parisa Norouzitallab, Thomas Van Leeuwen, Tim De Meyer, Gilbert Van Stappen, Yves Van de Peer, Peter Bossier

**Affiliations:** 1 Laboratory of Aquaculture & Artemia Reference Center, Department of Animal Sciences and Aquatic Ecology, Faculty of Bioscience Engineering, Ghent University, Ghent, Belgium; 2 Department of Plant Systems Biology, VIB, Department of Biotechnology and Bioinformatics, Ghent University, Ghent, Belgium; 3 Department of Data Analysis and Mathematical Modelling, Faculty of Bioscience Engineering, Ghent University, Ghent, Belgium; 4 Department of Plants and Crops, Faculty of Bioscience Engineering, Ghent University, Ghent, Belgium; 5 GNOMIXX, Boterbloemstraat 26, 9090 Melle, Belgium; 6 Coastal and Marine Sciences Institute, University of California, Bodega Bay, Davis, CA USA; 7 Department of Medical Technology, School of Health Sciences, University of Occupational and Environmental Health, Japan, Kitakyushu, Fukuoka, Japan; 8 Department of Pharmaceutics, Faculty of Pharmaceutical Sciences, Ghent University, Ghent, Belgium; 9 Laboratory for Immunology and Animal Biotechnology, Department of Animal Sciences and Aquatic Ecology, Faculty of Bioscience Engineering, Ghent University, Ghent, Belgium; 10 Centre for Microbial Ecology and Genomics, Department of Biochemistry, Genetics and Microbiology, University of Pretoria, Pretoria, South Africa

**Keywords:** Arthropod, Genome, Transcriptome, Assembly, Annotation, Extremophile, Salinity, Anoxia, *Artemia*, Brine shrimp

## Abstract

**Background:**

Brine shrimp *Artemia* have an unequalled ability to endure extreme salinity and complete anoxia. This study aims to elucidate its strategies to cope with these stressors.

**Results and discussion:**

Here, we present the genome of an inbred *A. franciscana* Kellogg, 1906. We identified 21,828 genes of which, under high salinity, 674 genes and under anoxia, 900 genes were differentially expressed (42%, respectively 30% were annotated). Under high salinity, relevant stress genes and pathways included several *Heat Shock Protein* and *Leaf Embryogenesis Abundant* genes, as well as the trehalose metabolism. In addition, based on differential gene expression analysis, it can be hypothesized that a high oxidative stress response and endocytosis/exocytosis are potential salt management strategies, in addition to the expression of *major facilitator superfamily* genes responsible for transmembrane ion transport. Under anoxia, genes involved in mitochondrial function, *mTOR* signalling and autophagy were differentially expressed. Both high salt and anoxia enhanced degradation of erroneous proteins and protein chaperoning. Compared with other branchiopod genomes, *Artemia* had 0.03% contracted and 6% expanded orthogroups, in which 14% of the genes were differentially expressed under high salinity or anoxia. One phospholipase D gene family, shown to be important in plant stress response, was uniquely present in both extremophiles *Artemia* and the tardigrade *Hypsibius dujardini*, yet not differentially expressed under the described experimental conditions.

**Conclusions:**

A relatively complete genome of *Artemia* was assembled, annotated and analysed, facilitating research on its extremophile features, and providing a reference sequence for crustacean research.

**Supplementary Information:**

The online version contains supplementary material available at 10.1186/s12864-021-07937-z.

## Background

Brine shrimp of the genus *Artemia* are small planktonic crustaceans found in hypersaline environments worldwide [1]. Their larvae (nauplii) are the most commonly used live larval food for marine fish and shellfish species reared in aquaculture [2, 3]. *Artemia* comprises six sexually reproducing, diploid species (*A. franciscana*, *A. persimilis*, *A. salina*, *A. sinica*, *A. tibetiana, A. urmiana* [4]) and several obligate parthenogenetic strains consisting of different clones and ploidies that cannot be grouped under one species [5]. Within each species, numerous local strains exist.

Halophiles are a subtype of extremophile organisms thriving in lakes and ponds with a salt concentration of at least 1.7% [6]. For reference, sea water has an average salinity of 35 g/L [7]. Until now, genomic research on halophiles has been limited to microbial organisms and a few eukaryotes, such as fungi, algae and land plants [8–11]. Because halophilic organisms possess stable enzymes that function under high salinity, efforts are ongoing to discover and utilize halophiles and their enzymes for biotechnology, including biofuel applications [10]. Little is known about the molecular mechanisms specific to animal halophiles. *Artemia* is one of the few known animal halophiles and is even considered an extreme halophile, tolerating salinities over 300 g/L [12]. Being obligate continuous filter feeders, they must excrete excess salt using specialised, developmental-stage-specific organs in nauplii (the salt gland), juveniles (the thoracic epipod) [13], and in adults (gut epithelium and the exopodite segments of the thoracopods) [14, 15].

Brine shrimps follow a typical life cycle. Under optimal conditions, adult females produce free-swimming instar I larvae (ovoviviparity) becoming adults within 2–4 weeks, whereas under stress (e.g., high salinity, low oxygen levels), they produce encysted gastrula embryos, named cysts (oviparity), that enter into diapause. These cysts remain viable for years, similar to plant seeds [16]. Cyst diapause is terminated only by strain-specific environmental stimuli (e.g., dehydration, freezing, exposure to low oxygen levels or light), leading to a quiescent state, the latter only terminated by hydration in oxygenated, temperate low salinity water (e.g. 30 g/L) and following a light trigger initiating the hatching metabolism. Hatched larvae grow into adults within 2–4 weeks.

Encysted embryos have a unique tolerance for anoxia, high doses of UV and ionizing radiation, thermal extremes, high and low atmospheric pressure (they hatch and develop in orbital spaceflight) [17] and desiccation [4, 18, 19] more than any other animal [19–21], partly owing to the presence of biological glasses [21] that contain trehalose and to late embryogenesis abundant (LEA) protein accumulation [22, 23]. Dry brine shrimp cysts are probably one of the most stress-resistant stages of all animals, outliving even tardigrades under ultra-high pressure [24]. Moreover, hydrated cysts have the ability to tolerate continuous, complete anoxia for up to 4 years, at physiological temperatures. The overall metabolism of anoxic embryos is brought to a reversible standstill, including the transduction of free energy and the turnover of macromolecules. Such an extraordinary stability is partly achieved in the cyst by deposition of massive levels of a small heat shock protein (p26) that acts as a molecular chaperone [25].

Knowledge of genes underlying these extreme *Artemia* phenotypes is of utmost interest and could contribute to making *Artemia* a promising model for stress response studies or host-microbial interaction studies. Some known examples of such genes are the osmoregulation gene *anterior pharynx-defective 1* [26], the cell cycle arrest termination gene *ribosomal s6 kinase* with direct applications in cancer treatment research [27], and genes coding for *Artemia* LEA proteins, enhancing desiccation tolerance in mammalian cells, thus enabling engineering of biostable dried cells [28]. However, published genomic resources for *A. franciscana* have been limited to the 15,822 bp mitochondrial genome sequence [29–31], an AFLP-based genetic map [32] and whole-transcriptome studies [33], including a whole transcriptome assembly [34].

Since the publication of the *Daphnia pulex* genome in 2011 [35], the first of now 46 sequenced and assembled crustacean genomes (Additional file 1: Assembly characteristics of all assembled crustacean genomes), only seven branchiopod genomes have been published. Here, a relatively complete, yet still fragmented genome assembly of *A. franciscana* is presented, representing a largely complete genic portion of the genome. The genome is used to describe the molecular pathways underlying some of the highly unique biology of *Artemia*, such as its salt and anoxia tolerance.

## Results

### Genome assembly and gene annotation

Illumina and PacBio DNA sequencing reads of an inbred *A. franciscana* resulted in a total average genome coverage of 53X by Illumina data (326 million Nextseq paired-end 150 bp reads and 1 million Miseq mate-pair 300 bp reads) and 11X by PacBio data (1,809,000 reads of on average 5531 bp). The genomic PacBio long reads and paired-end transcript data were used for genome scaffolding. After further gap-closing with the paired-end genomic data, a brine shrimp genome assembly of 849 Mb (see Additional file 2: Evolution of *Artemia* assembly quality metrics throughout the assembly steps) was obtained, thus achieving a 91% genome assembly completeness, compared to the 0.93 Gb *Artemia* genome size, as estimated by flow-cytometry [36]. Most currently available crustacean genomes are smaller than their estimated genome size, which is often ascribed to repeat contraction, or in some cases, mis-estimation of genome size (see Additional file 1). This includes the high-quality chromosome level genome assemblies of *Daphnia magna* and *Daphnia carinata* (assembly sizes are 42 and 51% of the respective genome sizes, see Additional file 1). The *Artemia* genome contains 26,057 contigs, further scaffolded into 20,887 scaffold genome fragments. The longest fragment in the assembly is 855 Kb long and the assembly shows a GC content of 34%, consistent with earlier estimations of 32% [37] and a more recent whole-genome estimation of 35% [36]. The N50 of the *Artemia* genome (scaffold N50: 112 Kb) was below the median N50 of crustacean genomes of similar genome sizes around 1 Gb (Additional file 1).

To support gene prediction, 15 million RNAseq read pairs from different mixed life stages of the inbred *Artemia* (whole body) were generated and assembled into a transcriptome (GC 37%; transcript number 76,045), with values similar to a previously published non-inbred *Artemia* transcriptome (GC 36%; transcript number 64,972) [34].

In the *Artemia* genome, gene structures were predicted with software packages EuGene and Augustus [38, 39], based on integrated data sets of *Artemia* RNAseq, EST and protein as well as arthropod RefSeq. 21,828 genes were identified and classified as non-repeat associated loci. Remarkably, genes were composed of, on average 4.41 exons per gene, with an intron length of 3458 bp, values standing out from other crustaceans. The set of high-confidence protein-coding gene loci was functionally annotated based on homology within NrProt, Genome Ontology (GO) and protein domains. A BUSCO analysis was applied to estimate the genome completeness (Additional file 3: BUSCO results for the *A. franciscana* genome assembly and annotation), suggesting that 75.5% of the 1066 BUSCO reference genes were present in the current annotation. The BUSCO reference genes are presumed to be generally present in arthropods, but are known to be biased towards model organisms (insects) [40], with crustaceans being underrepresented. This partially explains the missing BUSCOs.

None of the fragments in the assembly represented the complete mitochondrial genome (Additional file 4: BLAST results for mitochondrial genes in the *Artemia* genome). Since *Artemia* cannot be grown axenically to adulthood, some genomic fragments were identified as contaminants and removed from the *Artemia* genome (Additional file 5: Taxonomic groups of alien genomes identified in the *Artemia* genome): mainly bacteria (FCB group, Proteobacteria, Terrabacteria, unclassified bacteria), but also eukaryotes (Alveolata, Opisthokonta, Viridiplantae).

### Characterization of functional genome and repeat content

**Fig. 1:**
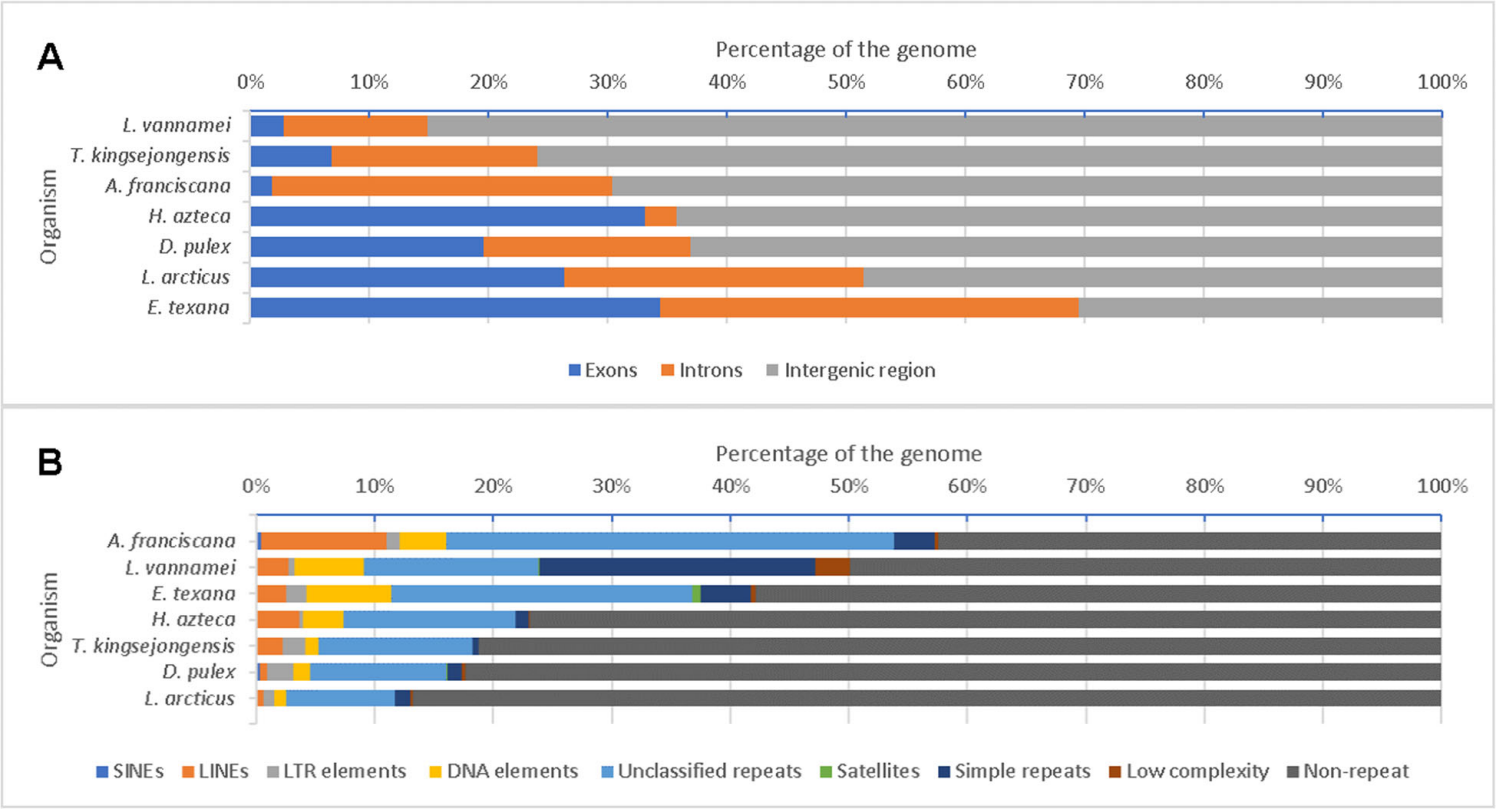
Exonic, intronic, intergenic and repeat content in crustacean genomes. Crustaceans shown: *A. franciscana*, *Litopenaeus vannamei*, *Eulimnadia texana*, *Hyalella azteca*, Tigriopus kinsejongensis, *Daphnia pulex* and *Lepidurus arcticus*. **A** Exonic, intronic and intergenic content. Relative coding and non-coding components of the whole *Artemia* genome and other arthropod genomes, based on component length (bp) compared to the total genome length (bp). **B** Repeat content in crustacean genomes. Relative repeat and non-repeat components of the whole *Artemia* genome and other crustacean genomes based on component length (bp) compared to the total genome length (bp)

The structural content of the *Artemia* genome was compared to other crustacean species (Fig. 1a). The *A. franciscana* genome showed 2.0% exonic space, similar to *Litopenaeus vannamei* (3%), while introns made up 29% of the genome, exceeding other arthropod genomes shown in Fig. 1, except *Eulimnadia texana* (30%). The relatively larger intron size can in part be attributed to the longer PacBio reads included in these genome assemblies [41]. Nevertheless, the intron content of *Artemia* was more than twice that of *L. vannamei*, which has a genome size twice that of *Artemia*.

Use of PacBio reads in the *Artemia* assembly process allowed to close and identify more repeats, as has been previously noted for other species [41]. Softwares RepeatMasker and RepeatModeler [42, 43] found a repeat content of 58% (Fig. 1b) in *Artemia*, higher than in *E. texana* and *L. vannamei*, in fact higher than in any other branchiopod or any crustacean. Interspersed repeats (SINEs, LINEs, LTR elements, DNA elements and unclassified interspersed repeats) spanned 53.6% of the *Artemia* genome. In addition, small RNAs represented 0.7% of the *Artemia* genome.

### Comparative genomics

To identify differences and similarities between the genome of *Artemia* from the order Anostraca and that of other branchiopod species from distinct taxonomic orders (Cladocera: *D. pulex*, Notostraca: *L. arcticus* and Spinicaudata: *E. texana*) a total of 16,912 orthogroups (gene families shared by different species) were considered in *Artemia* for expansion and contraction as compared to each studied species (more details in Methods section 2, Table 1).

**Table 1:** Species included in comparative genomics analysis

***Species***	***Taxonomic group***	***Proteome source***
*Artemia franciscana*	Crustacea: Branchiopoda: Anostraca	This study
*Daphnia pulex*	Crustacea: Branchiopoda: Cladocera	[44]
*Lepidurus arcticus*	Crustacea: Branchiopoda: Notostraca	[45]
*Eulimnadia texana*	Crustacea: Branchiopoda: Spinicaudata	[46]
*Tigriopus kingsejongensis*	Crustacea: Copepoda	[47]
*Litopenaeus vannamei*	Crustacea: Decapoda	[48]
*Hyalella azteca*	Crustacea: Malacostraca: Amphipoda	[49]
*Hypsibius dujardini*	Tardigrada	[50]
*Drosophila melanogaster*	Insecta	[51]
*Bombyx mori*	Insecta	[52]
*Tetranychus urticae*	Chelicerata	[53]

*Arthropod species included in comparative genomics analysis, their respective taxonomic group and the proteome source used for comparative genomics analysis*

**Fig. 2:**
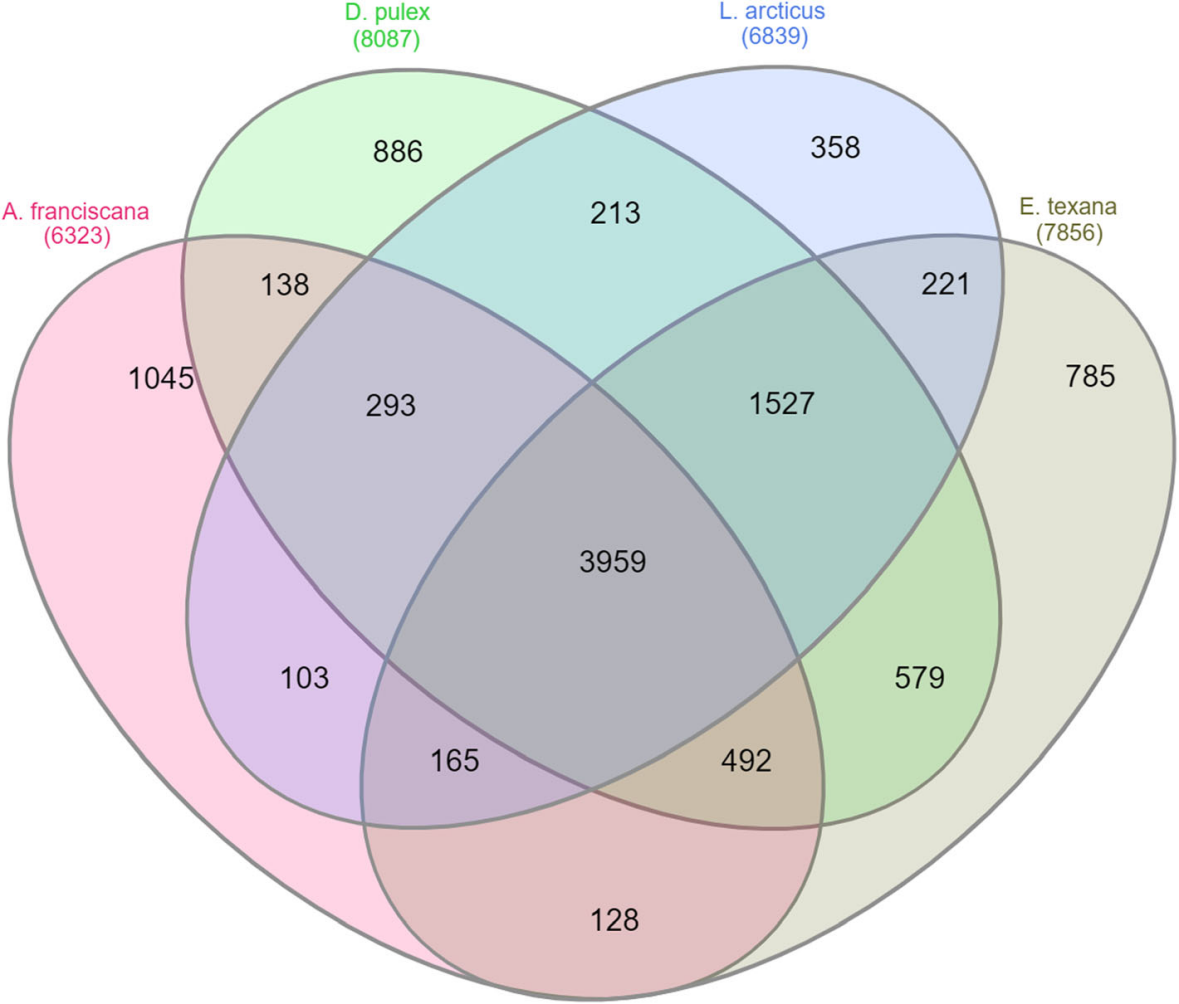
Comparative genomics between *Artemia* and other branchiopods. Venn diagram showing the number of orthogroups for each of the branchiopods *A. franciscana*, *Daphnia pulex*, *Lepidurus arcticus* and *Eulimnadia texana*

**Fig. 3:**
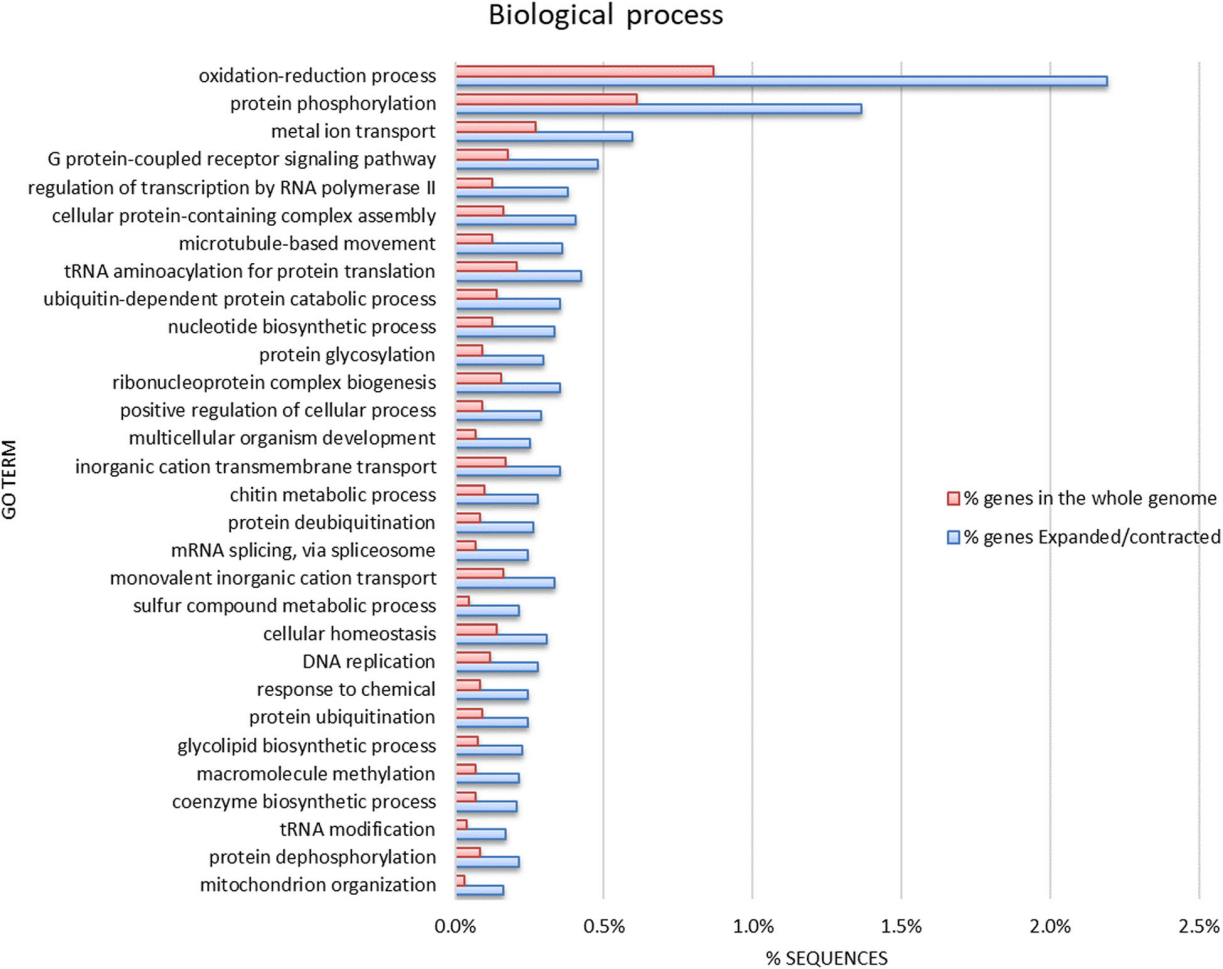
Significantly enriched gene ontology classes (GOs; Fisher’s exact test FDR ≤ 0.05) in *Artemia* compared to other branchiopods. The 30 most enriched GOs (biological process) in gene families expanded or contracted in *Artemia* (compared to other Branchiopoda), compared with GOs in the whole *Artemia* genome. Sorted from largest enrichment (top) to smallest enrichment (bottom). Enrichment was done by removing sequences present in both test set and reference set from the reference, but not from the test set, which makes the test more sensitive towards the test set, but can lead to zero values in the reference set

In total, 10,892 orthogroups were present in at least one branchiopod (Additional file 6: Expanded or contracted *Artemia* orthogroups compared to other Branchiopoda), of which 0.03% were contracted and 6% were expanded in *Artemia* compared with other branchiopods (Fig. 2). Of the genes in these orthogroups, 14% were differentially expressed under high salinity or anoxia (See Section “Functional genomics: salt and anoxia tolerance-specific genes”). The most enriched (Fisher’s exact test, FDR ≤ 0.05) gene ontology classes (GOs) in expanded or contracted gene families in *Artemia* compared to other Branchiopoda included amongst others (Fig. 3, Additional file 7: GO enrichment of *Artemia* compared to other Branchiopoda): redox maintenance, transport (metal ions, inorganic cations), protein metabolism and ubiquitination, protein folding and stability, cellular homeostasis, protein (de)phosphorylation, stress responses and finally, nuclear-encoded genes for mitochondrion organization, for cellular (dis)assembly or arrangement of the mitochondrion organelle. Many of these processes are involved in either salt or oxygen stress (See Section “Functional genomics: salt and anoxia tolerance-specific genes”). Several orthogroups, including Cytochrome b5-like heme/steroid binding domain superfamily (OG0011697) and the Phospholipase D family (PLD) (OG0009549) were uniquely present in *Artemia* and also in the extremophile *Hypsibius dujardini*, a member of the tardigrade phylum (Additional file 8: Expanded or contracted *Artemia* and *H. dujardini* orthogroups compared to other Arthropoda) and absent in other branchiopods.

### Salt and anoxia tolerance-specific genes

#### Differential expression analyses

RNAseq data from non-inbred *Artemia* was sampled and analysed under four different conditions: high salinity (200 g/L), low salinity (30 g/L), anoxia and normal oxygen levels. Gene expression of nauplii was compared under high (treatment) and low salinity (control) and gene expression of cysts was compared under anoxia (treatment) and normal oxygen conditions (control). For a detailed overview of the treatments, see Table 2, Methods section 3.2.

**Table 2:** *Artemia* sampling for RNAseq, including sampling time after treatment start and ARC cyst bank ID

Treatment	Life cycle stage	Sampling time (min)	Biological replicates per sampling time	Cyst ID	Species	Strain
Anoxia	cysts	60	3	ARC1767	*A. franciscana*	SFB
Hydration, normal oxygen	cysts	60	3	ARC1767	*A. franciscana*	SFB
High salinity (200 g/L)	instar I/II nauplii	15, 30, 45, 60	3	ARC1767	*A. franciscana*	SFB
Low salinity (30 g/L)	instar I/II nauplii	15, 30, 45, 60	3	ARC1767	*A. franciscana*	SFB

Sequence alignment with STAR resulted in an average of 50.3% uniquely mapped and 35.3% multimapped reads. This relatively low degree of uniquely mapped reads most likely reflects high sequence variability due to the use of non-inbred *Artemia* samples. Additionally, the inbred *Artemia* genome contained relatively short genes, possibly explaining the multimapped reads (Additional file 9: STAR mapping statistics for differential expression analysis in *Artemia*). As samples from different batches showed different mapping percentages (average difference 14%, *p* = 2.5E-13) and samples had been randomized over batches, we adjusted for batch effects as a factor during differential expression analysis (see Methods section 3). After data summary, on average 21.3% of mapped reads overlapped with annotated genes (Additional file 10: Summarization statistics for differential expression analysis in *Artemia*). Nevertheless, the transcriptome was sufficiently covered to perform differential expression analysis (19X; standard deviation = 7X).

In nauplii under high salinity (compared to low salinity), on average over all time points (15, 30, 45 and 60 min), 674 genes were significantly differentially expressed (adj. PVal< 0.05), of which 459 were upregulated and 215 downregulated.

In cysts under anoxia (compared to cysts with normal oxygen levels), 900 genes were significantly differentially expressed (adj. PVal< 0.05), of which 293 were upregulated and 607 downregulated.

The top 20 differentially expressed genes (DEG) with functional annotation, sorted by absolute log fold change (LogFC) under high salinity and anoxia can be found, including the adjusted *P* value (Adj. Pval) in Tables 3 and 4, respectively (full tables can be found in Additional file 11: Differentially expressed genes under high salinity and Additional file 12: Differentially expressed genes in *Artemia* under anoxia, respectively).

**Table 3:** The top 20 differentially expressed genes (LogFC-based) in *A. franciscana* under high salinity

Gene Id	LogFC	Adj. Pval	Functional annotation
artfr5078g00010	1.91	1.04E-05	*LEA6*
artfr4102g00020	1.62	6.94E-06	*catalase-like isoform X2*
artfr3493g00020	1.59	7.82E-05	*major facilitator superfamily domain-containing protein 12-like*
artfr401g00070	1.57	1.79E-07	*major facilitator superfamily domain-containing protein 12-like*
artfr498g00040	1.35	8.45E-05	*glutamate-cysteine ligase catalytic subunit isoform X3*
artfr8416g00010	1.26	6.09E-03	*trehalose-phosphate synthase 1*
artfr328g00050	−1.24	5.80E-04	*ATP-dependent DNA helicase pif1-like*
artfr363g00050	1.23	2.25E-05	*MFS-type transporter SLC18B1-like*
artfr1784g00020	1.17	1.94E-03	*chorion peroxidase-like*
artfr1161g00070	1.14	3.69E-03	*mitochondrial group 1 LEA protein precursor*
artfr4423g00020	1.12	2.83E-04	*cysteine proteinase precursor*
artfr1761g00010	1.10	2.10E-03	*organic cation transporter protein-like*
artfr6563g00010	1.08	1.77E-03	*patatin-like phospholipase domain-containing protein 2-like*
artfr1273g00010	1.07	1.12E-04	*cyclin-Y*
artfr537g00010	1.07	1.24E-05	*catalase homolog A*
artfr92g00010	1.07	3.85E-05	*LEA6*
artfr1351g00060	1.05	4.28E-09	*kruppel homolog 1*
artfr328g00060	−1.05	3.63E-03	*ATP-dependent DNA helicase pfh1-like*
artfr175g00080	1.05	6.58E-06	*dolichyl pyrophosphate Man9GlcNAc2 alpha-1,3-glucosyltransferase-like*
artfr8387g00020	1.04	1.03E-02	*Senescence-specific cysteine protease SAG39*

**Table 4:** The top 20 differentially expressed genes (LogFC-based) in *A. franciscana* under anoxia

Gene Id	LogFC	Adj. Pval	Functional annotation
artfr1563g00010	−2.86	1.42E-07	*TBC1 domain family member 23*
artfr690g00020	−2.78	6.92E-07	*putative DBH-like monooxygenase protein 2*
artfr1292g00020	−2.67	1.62E-08	*protein peritrophin-1-like*
artfr2964g00030	1.94	4.17E-06	*N-lysine methyltransferase SETD8-like*
artfr2663g00020	1.90	8.72E-07	*lysozyme 1-like*
artfr2034g00020	1.88	1.10E-07	*pentafunctional AROM polypeptide-like*
artfr427g00100	1.83	3.16E-06	*splicing factor pTSR1, putative*
artfr1672g00030	1.80	3.38E-06	*ABC protein, subfamily ABCH*
artfr3907g00020	1.72	8.70E-05	*protein bark beetle*
artfr503g00010	1.64	1.16E-07	*cytochrome P450 CYP18A1*
artfr1202g00050	−1.60	3.49E-03	*transcription initiation factor TFIID subunit 4*
artfr2964g00010	1.59	1.13E-08	*integumentary mucin A.1-like*
artfr188g00020	1.59	7.50E-14	*cuticle protein*
artfr1482g00010	1.59	2.21E-08	*aminopeptidase YwaD*
artfr316g00060	1.56	1.28E-14	*serine proteinase stubble*
artfr61g00120	1.51	3.16E-06	*adenosine monophosphate-protein transferase Fic*
artfr1404g00010	−1.49	5.42E-03	*heme-binding protein 2-like*
artfr10544g00020	−1.48	1.19E-02	*Beadex/dlmo protein*
artfr1487g00020	1.48	1.10E-04	*Cdk1/cks1*
artfr2018g00030	1.48	4.19E-05	*clathrin coat assembly protein*

To identify processes and pathways involved in salt and oxygen stress responses, significantly enriched GOs (Fisher’s exact test FDR ≤ 0.05) and pathways (Fisher’s exact test corrected for multiple testing, FDR ≤ 0.05) were determined in the list of significantly differentially expressed genes (*p* < 0.05) with software packages OmicsBox (Biobam, GUI software previously known as Blast2GO) and STRING v11.0, respectively. Only significantly overrepresented GOs and pathways (from here on more simply referred to as ‘enriched GOs’ or ‘enriched pathways’) obtained through these exploratory analyses are mentioned in the results and discussion sections.

#### Genes, GOs and pathways associated with high salinity

**Fig. 4:**
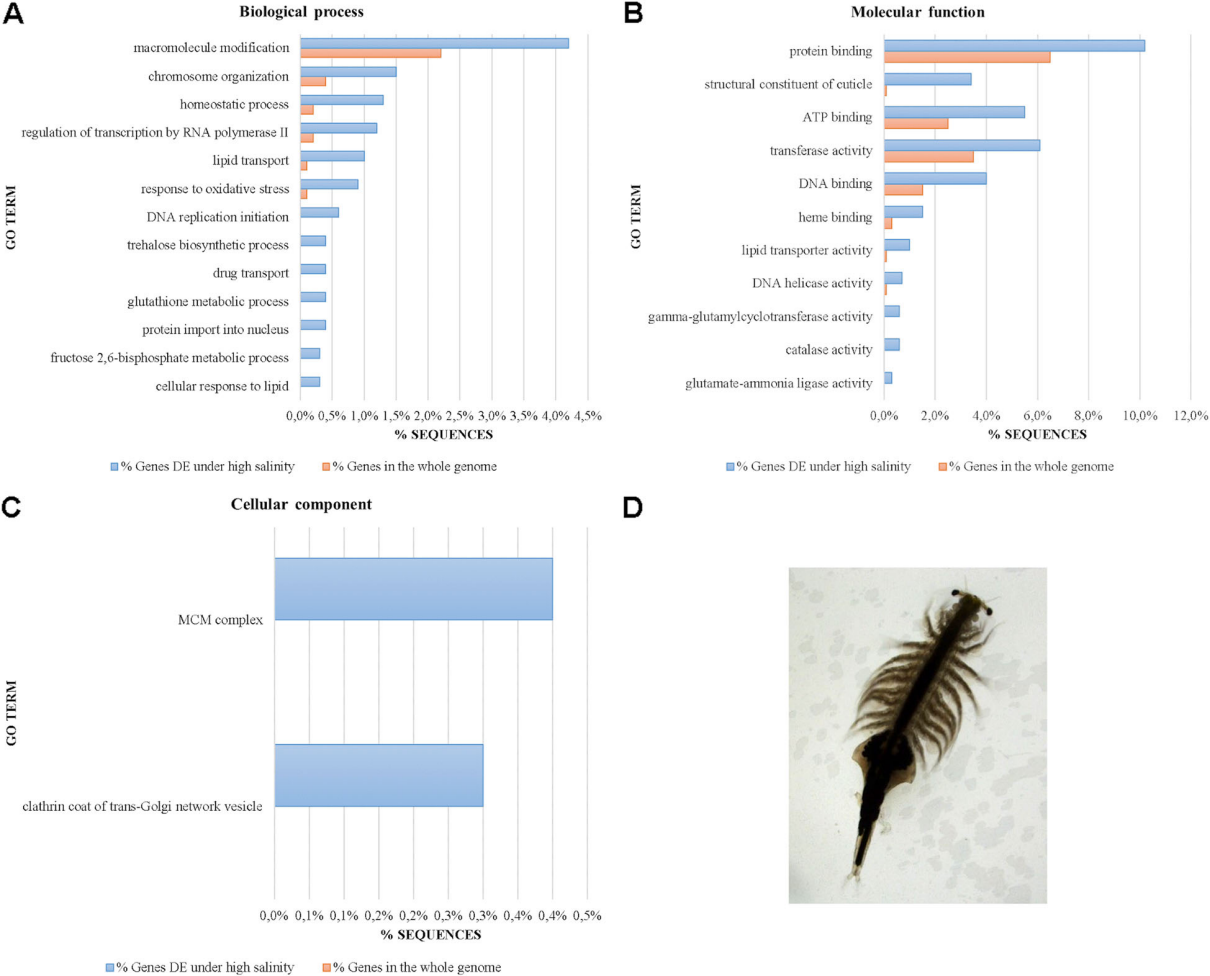
Significantly enriched gene ontologies (Fisher’s exact test, FDR ≤ 0.05) in the differentially expressed gene list under high salinity in *Artemia*. Biological processes (**A**), molecular functions (**B**) and cellular components (**C**) for genes differentially expressed under high salinity are shown. GO terms are sorted from the largest (top of the graph) to the smallest difference (bottom of the graph) between % sequences in the whole genome and in DE genes. Enrichment was done by removing sequences present in both test set and reference set from the reference only, but not from the test set, which makes the test more sensitive towards the test set, but can lead to zero values in the reference set. **D** Binocular image of an adult female *A. franciscana*

Under high salinity, several GOs for biological processes, molecular functions and cellular components were significantly enriched (FDR ≤ 0.05; Fig. 4; Additional file 13: GO enrichment in *Artemia* under high salinity) and this was also the case for several pathways (FDR ≤ 0.05; Additional file 14: Pathway enrichment in *Artemia* under high salinity). To facilitate further analysis, significant DEG, GO and pathway enrichment results under high salinity (Additional files 11, 13 and 14) were summarized into one file (Additional file 15).

##### DNA metabolism

Interestingly, *Artemia* under salt stress displayed significant downregulation of DNA repair genes (*nucleotide excision repair protein, ERCC-8*, *ERCC-5*, *DNA methyltransferase 1 associated protein 1*, see Additional file 15)*.* Additionally, genes responsible for telomere maintenance were significantly and highly downregulated, including *ATP-dependent DNA helicase pif 1-like* (Fig. 4b: DNA helicase activity, Table 3, Additional file 15), yet it remains to be determined whether this reflects genuine telomere biology or rather relates to the more general process of maintaining genome integrity. This gene was part of the significantly enriched GO “chromosome organisation” which also contained the following upregulated genes: two histone-lysine N-methyltransferases (*SETDB1-B, CG1716),* potentially involved in epigenetic transcriptional activation/repression, *sister chromatid cohesion protein PDS5* potentially also involved in DNA replication inhibition and *RNA polymerase-associated protein CTR9* involved in histone modifications (Additional file 15).

In contrast, *Artemia* displayed significant upregulation of several DNA replication licensing factors *mcm3*, *mcm3-like mcm4*, *mcm5-like*, *mcm6*, and *mcm6-b-like*. These genes are responsible for replication stress response [54] and are involved in the significantly enriched “MCM (mini chromosome maintenance) complex” (Fig. 4a, c, Additional file 15). Finally, the pyrimidine metabolism, DNA replication, spliceosome and the RNA transport pathways were also significantly enriched.

##### Oxidation-reduction and response to oxidative stress

Salt-stressed *Artemia* displayed a clear redox response, with all involved genes upregulated under salt stress. Significant GO enrichment was present in the processes “response to oxidative stress”, overlapping with significant GO enrichment of “catalase activity” with six highly upregulated catalases and a peroxidase (Table 3). Other significantly enriched GOs were the “glutathione metabolic process” with four upregulated glutamate-cysteine ligases and a *glutathione-specific gamma-glutamylcyclotransferase* as well as the “heme binding” process with upregulated *Cytochrome P450 315a1* and *(cyto) globin*, a known scavenger of nitric oxide or reactive oxygen species (Fig. 4a, b, Additional file 15).

##### Clathrin coat of trans-Golgi network vesicle (CCV)

In plants, the salt stress response with e.g. classically upregulated chloride ion transporter genes [55] is intensively studied at the genome-wide level, while in animals (fish, crustaceans), fewer studies are available and are mainly focused on lower levels of salt (up to seawater salinity) and on specific gene groups [56]. Endocytosis, a form of active transport in animals and plants, brings substances into the cell by surrounding them with a cell membrane, then budding off inside the cell to form a vesicle containing the ingested material. In plants, salt stress induces rapid clathrin-mediated endocytosis of NADPH oxidases to generate intravesicular reactive oxygen species (ROS) that presumably act as signalling molecules critical for salt stress tolerance [57]. During the first endocytosis steps in plants, clathrin-coated pits are internalized to form clathrin-coated vesicles (CCVs), which assemble at the trans-Golgi network [58]. In salmon eggs exposed to low salt levels (5 g/L), genes of the clathrin-mediated endocytosis signalling pathway are activated as well [59]. Under high salinity (200 g/L), *Artemia* showed significant GO enrichment for CCVs, with upregulated clathrin heavy chain genes (Fig. 4c, Additional file 15). Clathrin is the major protein of the polyhedral coat of CCV’s.

##### Protein processing in the endoplasmic reticulum and chaperones

**Fig. 5:**
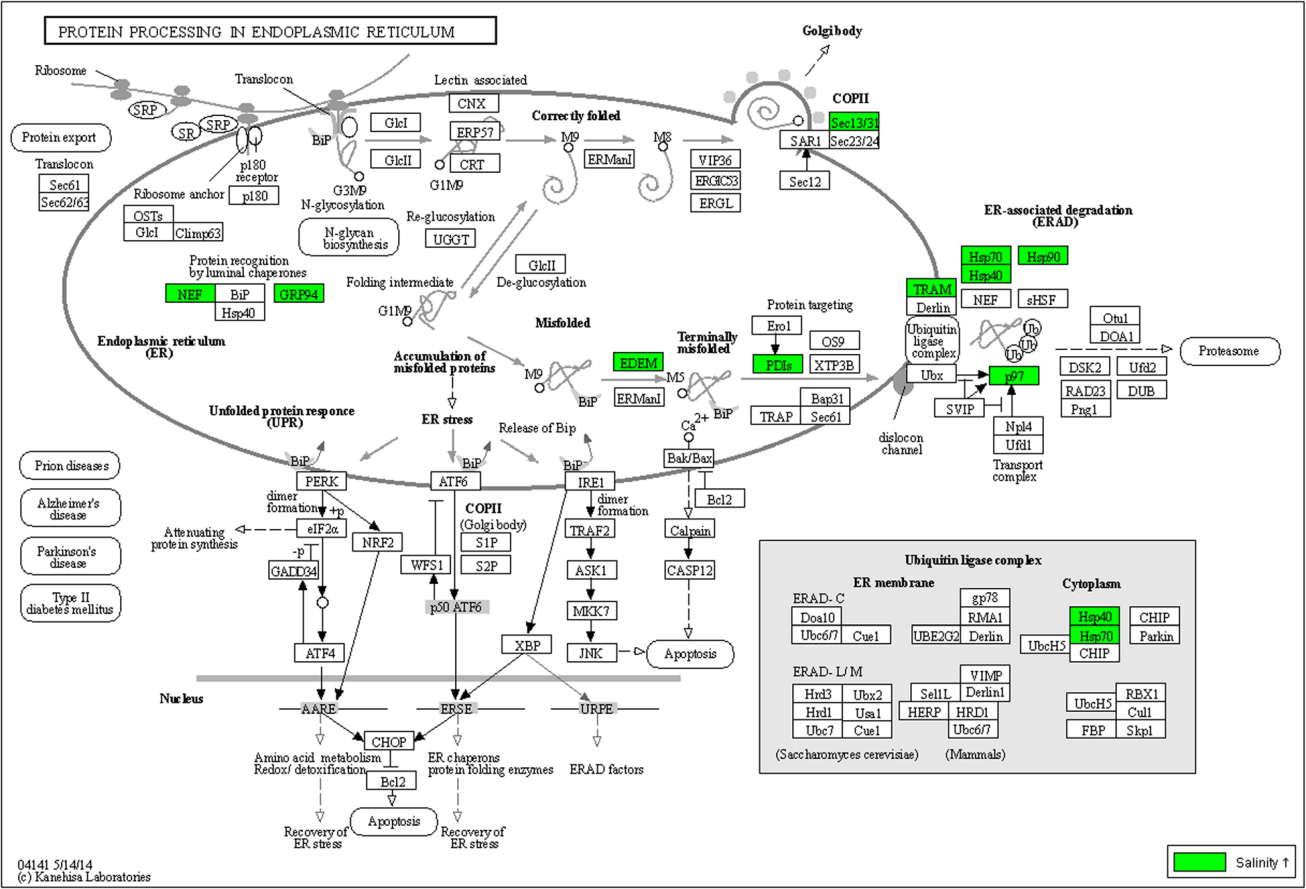
The pathway “protein processing in the endoplasmic reticulum”. This pathway is significantly enriched (Fisher’s exact test corrected for multiple testing, FDR ≤ 0.05) in the differentially expressed gene list in *Artemia* under high salinity. Significantly upregulated genes (*p* < 0.05) in *Artemia* are indicated on the *Daphnia pulex* pathway map dpx04141 from the KEGG database. No genes of this pathway were significantly downregulated in *Artemia*. Gene names and *Artemia* gene IDs are linked in Additional file 14 (tab sheets 1 and 2)

Molecular chaperones, many of which are heat-shock proteins (hsps), are an important class of molecules with various functions under stress. In *Artemia* and throughout the animal and plant kingdoms, they are a pivotal part of stress response [60]. The pathway for protein processing in the endoplasmic reticulum (KEGG pathway shown in Fig. 5) was significantly enriched and included upregulation of genes responsible for protein recognition by luminal chaperones (*nef*, *grp94*), recognition and targeting of terminally misfolded proteins (*EDEM, PDIs*), transport of misfolded proteins to the cytosol (*TRAM*, *p97*) and ER-associated protein degradation assisted by several heat shock proteins (*hsp40*, *hsp70 hsp90)*. Correctly folded proteins were likely transported by Golgi bodies, the formation of which is assisted by a protein transport protein (*Sec13/31*).

Other chaperone genes were found significantly upregulated as well (FDR < 0.05): four different *Hsp22*/*hsp20-like chaperones*, *heat shock protein 213 (with Alpha crystallin domain),* four different *DnaJ* genes, a *HSC70-like protein* and two *heat shock protein 90 like proteins* (Additional file 15).

##### LEA proteins

So far, LEA proteins have been known as osmotic stress or desiccation proteins [61]. Expression of three groups of late-embryogenesis abundant (LEA) genes has been found in *Artemia*: groups 1, 3, and 6 [62]. In this study, eight LEA genes were significantly (p < 0.05) upregulated under high salinity (See Additional file 11: Differentially expressed genes in *Artemia* under high salinity): 1) Five group 6 LEA genes, of which two were among the top 20 DEG (see Table 3), one of which (artfr92g00010) has been described before [62], 2) two putative group 1 LEA genes, one currently still broken in three parts, and 3) one group 3 LEA gene. In total, nine different LEA genes can be found in the *Artemia* genome, all of which are significantly upregulated under high salinity, except one (artfr2598g00030). While the current study shows that an important subset of the LEA genes in the *Artemia* genome is significantly upregulated under acute salt stress, aside from their previously known expression under desiccation conditions, [62] their potential function under other physiological conditions remains to be determined.

##### Metabolic pathways

Several genes involved in the trehalose metabolism were significantly upregulated: *trehalose-phosphate synthase 1*, responsible for the first step in trehalose synthesis, *facilitated trehalose transporter Tret1-like*, a transporter of trehalose, and a *trehalase-like* gene, probably catalysing the conversion of trehalose into glucose and glycerol.

Remaining significantly enriched pathways were the biosynthesis pathway of amino acids, including cysteine and methionine. The carbon metabolism pathway was also significantly enriched (KEGG pathway shown in Additional file 16), including the citrate cycle, and the metabolism of glyoxylates, dicarboxylates, nucleotide sugars, fructose and mannose.

An overview of all significantly enriched pathways can be found in Additional file 14 (FDR ≤ 0.05).

Additionally, salt stress significantly upregulated four major facilitator superfamily genes (Additional file 15), two of which were among the top 20 differentially expressed genes (Table 3). Some major facilitator genes described in plants are involved in small molecule transport, with gene deletions responsible for increased sensitivity to salt [63]. Hence, it can be hypothesized that *major facilitator superfamily* genes are involved in coping with the sharp salt increase from 30 to 200 g/L. The latter remains to be substantiated by establishing their physiological role with for instance RNAi experiments on *Artemia*, a methodology which can currently be used reliably in *Artemia* [64].

Interestingly, of the 832 DEG under high salinity in *Artemia*, 481 could not be ascribed to a GO class, indicating that some of the mechanisms involved remain to be unravelled.

#### Genes, GOs and pathways associated with anoxia

**Fig. 6:**
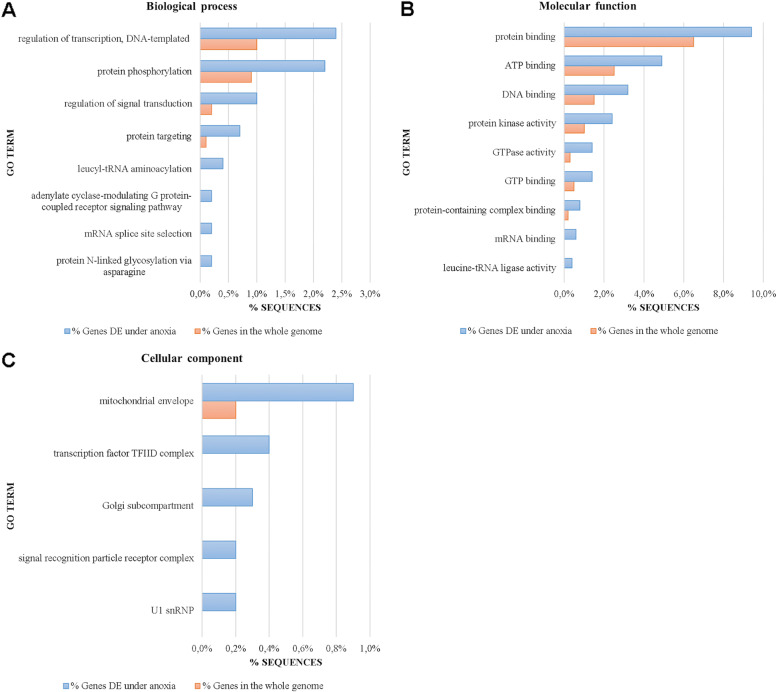
Significantly enriched gene ontologies (Fisher’s exact test, FDR ≤ 0.05) in the differentially expressed gene list under anoxia in *Artemia*. Biological processes (**A**), molecular functions (**B**) and cellular components (**C**) for genes differentially expressed under anoxia are shown. GO terms are sorted from the largest (top of the graph) to the smallest difference (bottom of the graph) between % sequences in the whole genome and in DE genes. Enrichment was done by removing sequences present in both test set and reference set from the reference, but not from the test set, which makes the test more sensitive towards the test set, but can lead to zero values in the reference set

Under anoxia, several gene ontology classes for biological processes, molecular functions and cellular components were significantly enriched (FDR ≤ 0.05; Fig. 6, Additional file 17: GO enrichment in *Artemia* under anoxia) and this was also the case for several pathways (FDR ≤ 0.05; Additional file 18: Pathway enrichment in *Artemia* under anoxia). To facilitate further analysis, significant DEG, GO and pathway enrichment results under anoxia (Additional files 12, 17 and 18) were summarized into one file (Additional file 19).

##### Mitochondrial function

Under anoxia, many genes involving mitochondrial function were significantly upregulated, indicating that enhancement of the mitochondrial metabolism may be involved in coping with anoxic circumstances: 1) genes responsible for mitochondrial protein translation (leucine--tRNA ligases), also part of the significantly enriched GO “leucine-tRNA ligase activity” (Fig. 6b, Additional file 19); 2) eight genes, present in the significantly enriched cellular component GO “mitochondrial envelope” (Fig. 6c, Additional file 19): genes respectively responsible for Cytochrome C oxidase assembly and maturation (*Cytochrome c oxidase assembly protein PET191*, *protein SCO1 homolog*), a gene required for mitochondrial cristae morphogenesis (*MSF1 protein)*, a mitochondrial intermembrane chaperone (*Tim13-B)*, a mitochondrial outer membrane import complex protein (*Metaxin-2*), a gene responsible for ATP production in the mitochondrial membrane (*ATP synthase epsilon chain*) and a proton pump in the mitochondrial envelope (*vacuolar H[+]vacuolar H + -ATPase*); 3) six different mitochondrially coded genes (*28S ribosomal protein S35, 39S ribosomal protein L19, 39S ribosomal protein L2, 39S ribosomal protein L22, mitochondrial, 39S ribosomal protein L27, 39S ribosomal protein L37*).

##### mTOR, the phagosome and autophagy

**Fig. 7:**
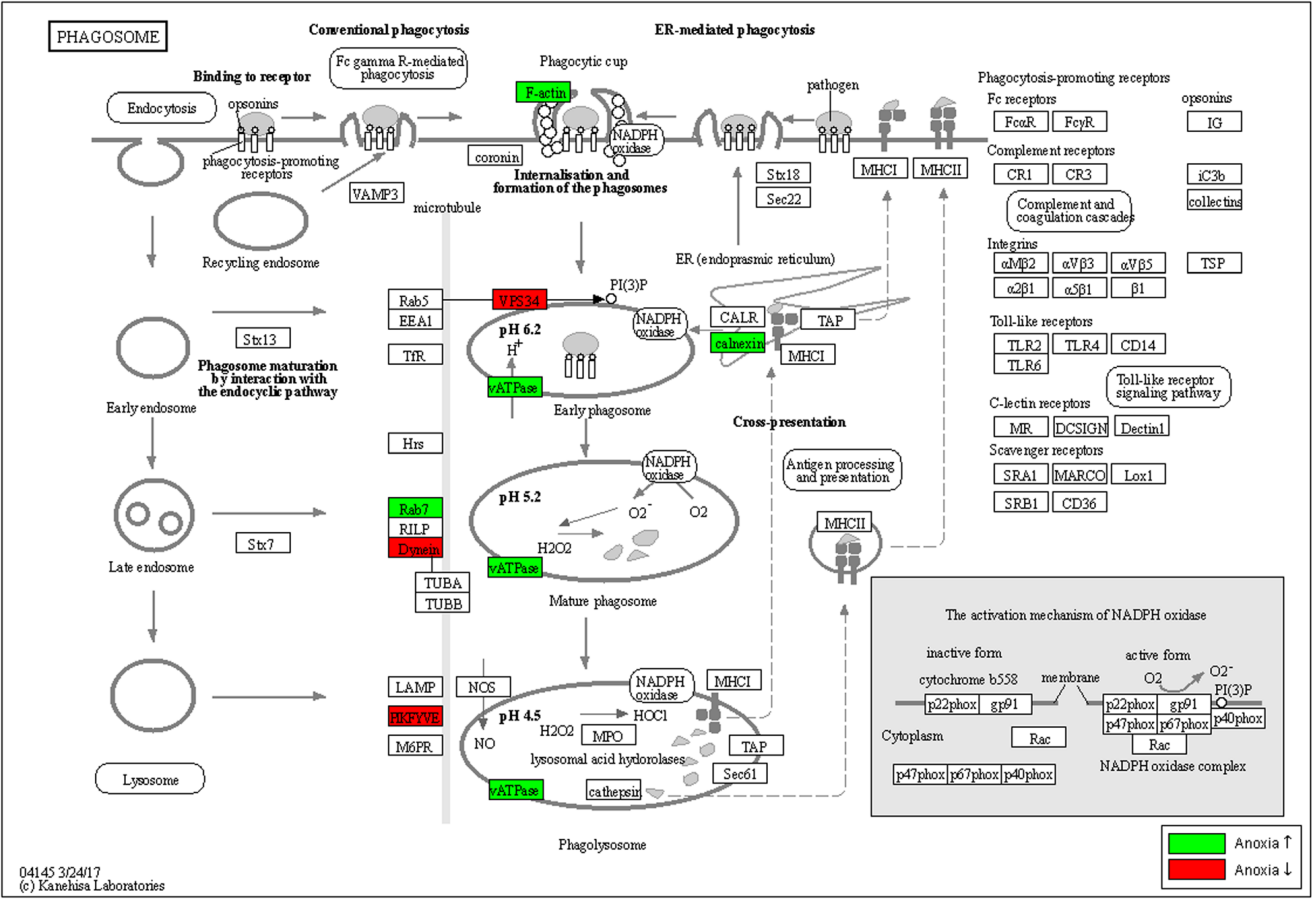
The pathway “Phagosome”. This pathway is significantly enriched (Fisher’s exact test corrected for multiple testing, FDR ≤ 0.05) in the differentially expressed gene list in *Artemia* under anoxia. Significantly up- and downregulated genes (*p* < 0.05) in *Artemia* are indicated on the *Daphnia pulex* pathway map dpx04145 from the KEGG database. Gene names and *Artemia* gene IDs are linked in Additional file 18 (tab sheets 1 and 2)

Hypoxia is known in humans to activate autophagy and to inhibit the *mTOR* gene, which in its turn further activates autophagy [65]. The mTOR signalling pathway was significantly enriched in *Artemia* under anoxia with 10 DEG (Additional file 19). The phagosome is responsible for tissue homeostasis. Under stress, it potentially functions as an autophagosome to degrade (damaged) cytosolic organelles for nutrients by fusing with lysosomes containing digestive enzymes. Under anoxia in *Artemia*, the phagosome pathway was significantly enriched (Additional file 19, KEGG pathway shown in Fig. 7), with gene sets partly overlapping with the significantly enriched autophagy pathway (Additional file 19).

##### Degradation of erroneous mRNA and proteins

The “mRNA surveillance” pathway, a quality control mechanism that detects and degrades abnormal mRNAs, was significantly enriched under anoxia in *Artemia* and contained only upregulated DEGs (Additional file 19).

The “N-glycan biosynthesis” pathway was also significantly enriched within DEGs under anoxia in *Artemia* (KEGG pathway shown in Additional file 20). N-glycans are critical for proper protein folding and quality control by chaperones in the endoplasmic reticulum (ER), a process now known as the “calnexin/calreticulin cycle” in the unfolded protein response and ER-assisted degradation [66].

As under high salinity, the pathway “protein processing in endoplasmic reticulum”, responsible for removing erroneous proteins in the ER, was significantly enriched under anoxia and included amongst others three significantly downregulated *hsp70* genes and a significantly upregulated *hsc70* gene. Furthermore, several other chaperone genes were significantly upregulated under anoxia: a mitochondrial *10 kDa heat shock protein*, known in humans to prevent misfolding and promote refolding of unfolded proteins generated under stress in the mitochondrial matrix, as well as *heat shock protein Hsp-12.2* and *Dnajc8* (Additional file 19).

##### DNA repair, transcription, RNA and protein transport and signalling

Two DNA repair pathways were significantly enriched in DEG, the “homologous recombination” pathway for reparation of double strand breaks and the “Fanconi anemia” pathway for reparation of interstrand crosslinks (Additional file 19).

In medical science, it is known that long-term hypoxia leads to transcriptional and/or translational downregulation of most DNA repair pathways, including DNA double-strand break repair, mismatch repair, and nucleotide excision repair [67]. This is potentially what happens in *Artemia* as this experiment measures the effects after an hour of complete anoxia, which is more extreme than a regular hypoxic state.

Finally, several pathways related to signalling, transcription and RNA and protein transport were significantly enriched in *Artemia* under anoxia: the “basal transcription factors” pathway responsible for transcription activation (KEGG pathway shown in Additional file 21), the “RNA transport” pathway containing only upregulated genes, the “spliceosome” and the “protein transport” pathways as well as the “phosphatidylinositol signalling” pathway (Additional file 19).

An overview of all significantly enriched pathways under anoxia is shown in Additional file 18 (FDR ≤ 0.05).

#### Genes associated with salinity and anoxia

Only 49 (3%) of all DEG were differentially expressed under both high salinity and anoxia, with 11 having no functional annotation. There were no common DEG among the top 20 DEG under high salinity and anoxia (Tables 3 and 4, respectively).

## Discussion

This is the first time that an Anostracan genome is assembled. Unique challenges were overcome for assembly and annotation of the *Artemia* genome. First, sequence variation was high, owing to the diploidy of *Artemia* and the need for several animals to generate sufficient DNA for long read DNA sequencing. To reduce sequence variation, the initial focus of the genome project was shifted from non-inbred *Artemia* to an inbred strain. Secondly, the unprecedented 58% repeat content in the *Artemia* genome led to a fragmented initial genome assembly. To our knowledge, *Artemia* has the highest repeat content ever recorded in crustaceans. Assembly fragmentation was greatly reduced on a limited budget by scaffolding the genome with a long-read DNA sequence coverage of 10X and N50 as well as gene integrity were further improved by scaffolding the genome with RNA sequence data. Thirdly, the *Artemia* genome had genes with relatively short exons and exceptionally long introns (3458 bp) compared to other arthropods. This was overcome by using tailor-made gene modelling for *Artemia*. As a result, and in spite of its unprecedented repeat content, unusual gene structure and limited budget, the *Artemia* genome assembly had a GC content of 34% in correspondence with literature, a genome assembly contiguity of the same order of magnitude as other similar-sized crustacean genomes (scaffold N50 of 112 Kb), and a 21,828 gene genome annotation, containing 75.5% of the BUSCO reference genes found in other arthropods. While BUSCO analysis results are often not included in crustacean genome publications, this is in a similar range as the 88% BUSCO reference genes found in the *E. texana* genome, a highly contiguous crustacean assembly considered as a new standard for crustacean genomes [46].

*Artemia* has been described as an extremophile, with nauplii and adults able to survive and thrive at extreme salinities in lakes and saltworks and producing cysts able to survive anoxic conditions. 

Genome-wide research on halophiles has so far largely been limited to microbial organisms and a few eukaryotes, such as fungi, algae and land plants [8–11]. As far as we know, this study is the first genome-based study of a halophilic animal. Recently, a transcriptome-based differential expression analysis identified salt stress responses in *Artemia* involving *EIF* (eukaryotic initiation factor), *Heat shock 70 kDa protein cognate*, the chitin metabolic pathway, DNA repair, ubiquitination, cell cycle arrest (*La-related protein*), lipid metabolism and (metal) ion transport [34]. The current study, while genome-based and more extensive, largely confirms the findings of the previous transcriptome analysis: 1) *EIF* was involved (significant upregulation of genes *NAT1A* and *EIF4AIII*), 2) a *HSC70-like* gene was significantly upregulated, 3) DNA repair-related genes were significantly downregulated, 4) the gene *La-related protein 6A* was significantly upregulated, and 5) the GO terms “cellular response to lipid”, “lipid transporter activity” and “heme binding” as well as the cellular component GO “structural constituent of cuticle” were significantly enriched.

All genes related to “structural constituents of the cuticle”, including several cuticle proteins were downregulated in *Artemia* under high salinity. Under salinities as high as 200 g/L, growth in *Artemia* is substantially slowed down due to its increased energy needs for osmoregulation, whereas at lower salinities (e.g. the 30 g/L control in our experiment), the brine shrimp larva moults every few hours into a subsequent developmental stage until maturity [68]. Moulting involves many regulatory mechanisms and related genes, including regulation of cuticle-related enzymes such as chitinase and structural proteins of the cuticle [69]. Transcriptomic analysis in crustaceans *Portunus trituberculatus* [70]; *Euphausia superba* [71] and *L. vannamei* [72] at different stages of the moulting cycle showed upregulation of structural constituents of the cuticle during the moulting cycle, with upregulation of cuticle genes during pre-moult and downregulation during inter-moult [12]. Exposure of the *Artemia* nauplii to high salinity thus corresponds with a prolonged state of inter-moult.

For several biological processes previously reported in plant salt stress, similar processes were found in *Artemia* and these were significantly enriched in the DEGs under the *Artemia* salt stress response in this study. In plants, salt stress causes genotoxicity in which DNA alteration/damage can arise as a consequence of errors in DNA replication and DNA repair [73], which also seems to be the case in *Artemia*. In plants as in *Artemia,* MCM family genes show salt stress-responsive behaviour [74] and the phagosome is also an enriched gene ontology feature, as found in the salt-tolerant plant *Suaeda salsa* under high (30 g/L) salinity [11].

In halophytic plant species, there is strong evidence in favour of clathrin-dependent vesicular transport mechanisms of Na^+^ and Cl^−^ ions as a feature of salt tolerance. During endocytosis, parts of the plasma membrane enclose extracellular substances in vesicles eventually fusing with lysosomes and vacuoles to isolate the plant from the excess salt. During exocytosis, these vesicles export excess ions via plant salt glands [75]. Interestingly, under high salinity, *Artemia* showed significant GO enrichment of processes in the clathrin-coated vesicles, including *clathrin* upregulation. *Clathrin* was also part of an expanded gene family in *Artemia* compared to other non-extremophile branchiopods. Hence, it appears possible that *Artemia* would transport salt via clathrin-dependent vesicular transport (endocytosis) for final salt excretion (exocytosis). It remains to be established if exocytosis is ongoing via the *Artemia* salt gland [76], which is still present at the sampling time points chosen in this study and remains present, at least until larval stage instar III, after which it is resorbed in adulthood.

Protein processing in the endoplasmic reticulum and chaperones, both found to be important *Artemia* salt stress responses in this study, play a key role in targeting and degrading misfolded proteins by ATP-dependent heat shock proteins. Both the use of ATP-dependent *HSP* and, potentially, endo-and exocytosis of salt, are energy-intensive processes, which may explain the enhanced carbon metabolism in *Artemia* under high salinity.

Salt-stressed *Artemia* also showed a redox response similar to that observed in plants [77] and in the intertidal flatworm *Macrostomum lignano* [78], all perturbed in redox homeostasis under salt stress. Salt stress generally induces reactive oxygen species (ROS), causing oxidative stress-induced damage to cellular components, which can be neutralized by antioxidants [79]. Catalases and peroxidases, heme enzymes involved in ROS metabolism, were upregulated in *Artemia* under salt stress, suggesting that they were directly responsible for ROS control. Transition metals are known to function as balancing factors and signalling for ROS in plants [80]. Transition metal binding genes were also upregulated under salt stress in *Artemia*. Salt stress most likely induced a strong oxidative stress in *Artemia*, which was then balanced with transition metal binding proteins.

LEA proteins were part of an expanded gene family in *Artemia* compared to other branchiopods (Additional file 6; OG0007033, 9 genes including one with “seed maturation protein” motive). They are important for cellular protection during desiccation in eukaryotic cells by e.g., stabilizing membranes [81] and were upregulated in *Artemia* under high salinity, potentially to protect cells from desiccation. Such a high number of LEA genes has also been described in tardigrades (with variable numbers depending on the species), where they contribute to desiccation tolerance [82, 83]. On the other hand, homologs of other tardigrade-specific proteins involved in tardigrade desiccation tolerance (such as CAHS, SAHS, and MAHS) [84], were not present in the *Artemia* genome (results not shown). Tardigrades and *Artemia* thus share some common strategies relative to osmotic stress and/or desiccation (e.g., ROS mitigation, trehalose production, HSP induction, LEA), but they probably also harbour species-specific genes.

The genes in the hypoxia-inducible factor (*HIF*) pathway are known master regulators of oxygen sensitivity and distribution inside cells, and are therefore considered highly conserved in most animal groups. Homologs of the *Marsupenaeus japonicus* hypoxia-inducible factor (*HIF-1α* [85]) were identified in the *Artemia* genome (results not shown) but were not differentially expressed under anoxia in this study. Homologs of *VHL* (Von Hippel-Lindau tumor suppressor), regulating *HIF-α* degradation and *FIH-1*, hydroxylating a specific residue of HIF-α under normoxic conditions, were both absent in the *Artemia* genome. Within the Crustacea, even if the *HIF* pathway can be found in several species, it has been lost in other crustaceans, such as the intertidal *Tigriopus californicus*, where alternative hypoxic stress response mechanisms are believed to play a major role, such as genes involved in cuticle reorganization and ion transport [86]. It is possible that *Artemia* has partially lost its *HIF* pathway and uses an alternative hypoxia response through cuticle reorganization and ion transport. This seems plausible, as *Artemia* is a halophile and the gene *cuticle protein* was one of the top 20 differentially expressed genes in *Artemia* under anoxia. Moreover, it could be that the *HIF* pathway is involved in regulating low-oxygen (hypoxic) conditions, but not full anoxia such as *Artemia* was exposed to in this study.

In the freshwater crayfish *Orconectes virilis* under severe and prolonged anoxia (5–20 h), cAMP-dependent protein kinases strongly decrease and phosphatase activity is strongly increased [87], while hypoxia inhibits *mTOR*, activating autophagy [88]. In *Artemia*, many protein kinases were up- and downregulated, while most (protein) phosphatase genes were upregulated (Additional file 19). It is possible that increased phosphatase activity plays a role in metabolic adjustment of *Artemia* to prolonged anoxia. The *mTOR* signalling and autophagosome pathways were significantly enriched in *Artemia* under anoxia as well. It seems that under anoxic stress in *Artemia*, the *mTOR* pathway activates the autophagosome pathway to recycle (damaged) cytosolic organelles for nutrients.

Mitochondria are major oxygen consumers and a potential source of ROS. In response to hypoxia, the mitochondrial metabolism often becomes more efficient [89]. In *Artemia*, mitochondrial function was clearly enhanced with significant upregulation of genes responsible for ATP production in the mitochondrial membrane, cristae morphogenesis, mitochondrial transport and intermembrane chaperoning.

In this study, comparative genomics showed that *Artemia* had the highest number of unique gene families compared to other Branchiopods, containing gene expansions in domains important for salt stress tolerance, such as redox response and endocytosis-related mechanisms, as well as for anoxia tolerance, such as protein phosphorylation. The PLD gene family, while its genes were not differentially expressed under high salinity or anoxia in *Artemia*, was expanded compared to other, non-extremophilic branchiopods. The PLD gene family consists of lipid metabolic genes that degrade membranes, thus triggering a signalling cascade molecule influencing the mTOR pathway towards survival or inflammation responses, thus initiating proliferation, transcription, cytoskeletal organization and NADPH oxidase and vesicle formation for exo- or endocytosis [90]. PLD genes, also found in tardigrades, were first described in plants, in which they are typically activated by a wide variety of stressors: cold-, drought- and salinity-stress, wounding, pathogenic elicitation and phosphate starvation [91]. 

While many mechanisms underlying the salt stress and the anoxia responses of *Artemia* were identified, still 32% of DEG under salt stress and 21% under anoxia had no known function. Since functional gene annotation is based on homology with other organisms, it is possible that mechanisms unique to *Artemia* are still hidden within these unknown genes**.**

*Artemia* has been used in the study of host-microbial interactions (e.g. *Vibrio* infection), uniquely allowing *Vibrio* challenge tests under gnotobiotic conditions [92]. *Artemia* is also, through its short generation time and large population size, susceptible to truncation-based genetic selection. Cysts generated in such experiments can be stored and hatched simultaneously, allowing for common garden experiments [93]. Moreover, parthenogenetic strains of *Artemia* are instrumental in the study of transgenerational phenotype inheritance and hence in the research of epigenetic processes [93]. Finally, *Artemia* shows a relatively large similarity with the recently published *L. vannamei* genome (2.45 Gb [94];), containing over half of its orthogroups, including typical *L. vannamei* immunity-related genes, without expansion or contraction (results not shown). It is anticipated that with both annotated genomes currently now available, it will be possible to fully explore the potential of *Artemia* as a model species for commercial aquatic crustaceans in the fields of host-microbial interaction, genetics and epigenetics.

## Conclusions

Here, we present the genome assembly and annotation for an inbred *A. franciscana,* as well as comparative genomics with other animals and functional genomics under high salinity and anoxia. Compared with other branchiopods, the *Artemia* genome has many contracted and expanded gene families involved in different strategies for coping with stressors including high salt and anoxia. This means that the differences between *Artemia* and other branchiopods can probably be largely attributed to the stress coping abilities of *Artemia*. Under high salinity, *Artemia* potentially uses plant-like, energy-intensive strategies to enclose excess salt in the cell, as well as DNA repair, extensive molecular chaperoning, redox and osmotic balancing, and LEA proteins and trehalose against desiccation. Under anoxia, mitochondrial function was enhanced, while the mTOR pathway activated the autophagy pathway, potentially for recycling of damaged cell components as nutrients.

## Methods

All bioinformatics up until genome characterization with DBG2OLC were performed on the VIB-PSB Biocomputing Linux cluster, all subsequent steps were performed on the HPC-UGent, supported by the Flemish Supercomputer Center.

### Genome assembly

#### Sampling, DNA extraction, sequencing and read processing

##### Sampling

Inbred *A. franciscana* Kellogg, 1906 (Great Salt Lake (GSL), Utah, USA) were reared at the Department of Medical Technology, University of Occupational and Environmental Health, Kitakyushu, Japan: a 48 generation inbred female parent (sample #421, as shown in Nambu et al. under “GSL2” [95]) and a 62 generation inbred male parent (sample #870, as shown in Nambu et al. under “GSL2” [95]), were crossed to produce F_2_ progeny cyst specimens kept at 4 °C, in 20 g/L salt. Cysts of “Specimen 9” were hatched (28 °C, salinity 10 g/L), the nauplii were harvested. Considering the hatching time frame, these nauplii should be instar I or II, which are difficult to separate during the hatching process. Nauplii were reared until sexual maturity in aerated seawater with added sea salt (Instant Ocean® USA, 28 °C, final salinity 70 g/L) and fed with microalgae *Tetraselmis suecica*. Mature animals were rinsed with sterile distilled water, the phenotypic sex of each individual offspring was determined visually and females were selected for further processing. Females were favoured since they are the heterogametic sex, thus ensuring sequencing of both the Z and the W chromosomes. For gut evacuation before DNA extraction, the females were kept overnight in a cellulose solution (1.5 g/L, type 20, Sigma-Aldrich USA) [96], followed by removal of the brood pouch. The animals were stored individually at − 20 °C.

##### DNA extraction

DNA was individually extracted from seven female individuals grown from Specimen 9 cysts according to a modified CTAB-method for shrimp tissue [97]. Briefly: each sample, homogenized in 150 μl of CTAB buffer with 3 μl RNAse A (from the Wizard® Genomic DNA Purification Kit, Promega USA) was kept for 30 min at 37 °C, then re-incubated 30 min in CTAB buffer (0.25% v/v 2-mercaptoethanol). After homogenization, 750 μl of extra CTAB buffer was added and the mix was left at 25 °C for 30 min and PCA solution was added (600 μl, 25:24:1 phenol/chloroform/isoamylalcohol). After centrifugation, 800 μl of the upper aqueous phase was supplemented to 600 μl of CA solution (24:1 chloroform/isoamylalcohol) and the mix was homogenized. To 700 μl of the upper aqueous phase, 630 μl of isopropanol was supplemented. The mix was incubated for 1 h at − 70 °C. After centrifugation, the pellet was washed with 600 μl of ethanol 70%, air-dried in a 60 °C oven and resuspended in 20 μl of sterile distilled water. DNA quality and concentration were assessed on an agarose gel and by NanoDrop™ spectrophotometer (Thermo Fisher Scientific USA).

DNA from inbred females served as the template for PacBio (Pacific Biosciences USA) and Illumina (USA) sequencing technologies.

##### Sequencing and read processing: Illumina

DNA from one female individual (sample name F2) from Specimen 9 was sequenced with several technologies, as described below.

One library (500–800 bp) was prepared for the Illumina sequencing: Illumina NextSeq library prep was done with the NEBNext® Ultra™ DNA Library Prep Kit for Illumina® (New England Biolabs, USA). The library was paired-end sequenced with Illumina NextSeq 500 High 300 Medium technology (read length 150 bp, intended output 35X, NucleomicsCore, Leuven) and with Illumina MiSeq 600 v3 technology (non-stitched, read length 300 bp, intended output 20X, NucleomicsCore, Leuven). Read quality was tested using the software FastQC v0.11.2 [98]. Illumina PE reads that contained overrepresented sequences of adapters (TruSeq Adapter, Index 1, Illumina universal adapter [99] were adapter-trimmed with CLC assembly Cell v4.0.13 (Qiagen, Germany [100]), then quality-trimmed with CLC assembly Cell v4.0.13 (quality score minimum 20, minimum length of PE reads, 36 bp) to produce a paired-end and a single-end output [100]. Read quality was again tested using the software FastQC v0.11.2 [98].

##### Sequencing and read processing: PacBio

A total of 10X intended sequencing output by PacBio technology was generated: 3–20 kb DNA from four female individuals from Specimen 9 (AFRFI4, AFRFI7, IF4, IF13) was sequenced on 8 SMRT cells using PacBio RS technology with 2X intended coverage (NucleomicsCore, Leuven), and 10 kb DNA from two other Specimen 9 females (AFRIF-19-62-III-PACB, AFRIF-63-241-IV-PACB) was sequenced on 12 SMRT cells using PacBio RSII technology with 8X intended coverage (BaseClear, Netherlands). The data collected from the PacBio RS instrument were processed and filtered using the SMRT Analysis software suite (PacBio, USA). The Continuous Long Read (CLR) data were filtered by Read-length (> 35), Subread-length (> 35) and Read quality (> 0.75).

Read quality was tested using the software FastQC v0.11.2 [98]. PacBio reads error correction was performed using the PE and SE trimmed inbred Illumina reads with the software LoRDEC v0.5 [101] (lordec-correct -k 21 -s 2). Read quality was again tested using the software FastQC v0.11.2 [98].

#### Hybrid assembly

Previously to this publication, a non-inbred *A. franciscana* (SFB x VC) genome (SUB5823124) was assembled at the ARC as a side product of a bulked segregant analysis [36], out of paired-end (insert size 200–500 bp) and Cre-lox mate pair [102] Illumina sequencing (insert size 3 kb, NXTGNT, Belgium, Scripps Research Institute, USA) of a mixed-sex pool of adult *Artemia*. Since it was constructed from Illumina sequences of a pooled sample of non-inbred diploid animals, and the genome contained many repeats (44%), heterozygosity was high at almost 4 M SNPs in the 1310 Mbp genome assembly, redundancy was 41% compared to the published genome size of 0.93 Gb [32], and the assembly was still fragmented in 176,667 scaffolds, resulting in a fragmented annotation of 188,101 genes [36].

Therefore, the new inbred *A. franciscana* (GSL) genome described in this publication was subsequently assembled, based on Next-Generation Sequencing (NGS) data from one female and scaffolded with PacBio data from six additional females. Various assemblies were generated using different combinations of software and subsets of data. The best assembly was selected according to scaffold N50 and assembly size. The foundation of the chosen assembly was assembled using the SPAdes genome assembler v3.10.0 (read error correction 1 iteration, assembling mode, multi-cell, parameters: -k 21,33,55,77,99,127 ---careful) [103] with 3 input read libraries: 1) the paired-end result of the trimming (orientation: fr, interlaced, miseq or nextseq), 2) the single-end result of the trimming, 3) the single-end PacBio reads.

#### Scaffolding

The SPAdes genome assembly result with K-mer size 127 was scaffolded using three different scaffolding software packages:

1. DBG2OLC v20150519 [104] was used to scaffold the genome with the inbred *Artemia* DNA sequencing reads: a first scaffolding round with the inbred Illumina PE and SE data, a second scaffolding round with the inbred PacBio data, and a third scaffolding round with the inbred Illumina PE and SE data, according to the following command lines (italic):

- *dbg2olc k 50 KmerCovTh 0 MinOverlap 70 LD1 0 PathCovTh 3 Contigs contigs.fasta f illumina_PE.fastq f illumina_SE.fastq.*
- *dbg2olc MinLen 100 k 17 KmerCovTh 2 MinOverlap 10 AdaptiveTh 0.001 LD1 0 RemoveChimera 0 Contigs. /DBG2OLC_Consensus.fasta f pacbio.fastq.*
- *dbg2olc k 50 KmerCovTh 0 MinOverlap 70 LD1 0 PathCovTh 3 Contigs. /backbone_raw.fasta f illumina_PE.fastq f illumina_SE.fastq.*

2) L_RNA_scaffolder v20141124 [105] was used to scaffold the genome with all transcripts from the non-inbred *A. franciscana* transcriptome [34] and the following command line:

- *blat /dev/null /dev/null -makeOoc = 11.ooc -repMatch = 1024, blat -t = dna -q = rna -ooc = 11.ooc -fine -noHead, sh L_RNA_scaffolder.sh -d. -l 0.9 -p 0.9 -e 100,000 -f 1.*

3) SSPACE_Basic v2.1.1 [106] was used for re-scaffolding with the inbred Illumina PE reads and the following parameters:

*-× 0 -g 3 -k 3.*

#### Gap filling and polishing

Gaps in the scaffolded genome were filled with GapFiller v1.10 [107] using the inbred Illumina PE reads (−m 30 -o 2 -r 0.7 -n 10 -d 50 -t 10 -g 0).

The gap-filled genome sequence was polished with 10 iterations of BWA v0.7.15 [108]. mapping and Pilon v1.21 [109] polishing to remove errors introduced by the DBG2OLC step with PacBio data (other than default parameters are: --mindepth 3 --fix bases). Pilon was run with inbred PE Illumina gDNA reads that were themselves, error-corrected using the software BrownieAligner [110]. To increase coverage at transcribed loci, an inbred RNA-seq library, dedicated to annotation was included (whole animal, MiSeq reads PE 300 nt). The Pilon corrections performed were limited to SNP, ambiguous nucleotide resolving, and small insertion/deletions (−-fix bases).

#### Gene annotation

The genome was structurally annotated using several data sources, including a new inbred *Artemia* transcriptome assembled for that purpose.

#### Inbred transcriptome

##### Sampling

F_2_ progeny from the inbred *A. franciscana* (GSL, section 1.1) was further interbred in one tank for 2 months during which cysts were continuously harvested, after which they were stored in saturated NaCl-brine at 7 °C. These cysts were transferred to a Petri dish (28 °C, salinity 25 g/L Instant Ocean® USA) and sampled after 0 h (quiescent), 4 h, 8 h, 12 h, 16 h and 20 h. After 24 h, a sample of nauplii was taken. The remaining animals were further reared (aerated seawater with added Instant Ocean® USA, 28 °C, final salinity 70 g/L using *T. suecica* as feed. After 36 h, a sample of nauplii was taken, and the remaining animals were reared under the same conditions until sexual maturity. One adult male and one adult female were sampled. All samples were rinsed with autoclaved mQ on a clean sieve, put in a 1.5 ml sterile Eppendorf tube with a sterile spatula, flash-frozen in liquid nitrogen and kept at − 80 °C.

##### RNA extraction

For each sample, RNA was extracted by a combined method with TRIzol (Invitrogen, USA) and the RNeasy Mini Kit (Qiagen, Germany) as described previously [34], except that each freeze-dried sample was homogenized with 0.5 mm glass beads in 500 μl of TRIzol (1 min homogenizing, 30 s rest, 1 min homogenizing), each centrifugation step was performed at 4 °C, and DNA contamination was removed with the gDNA Eliminator column from the RNeasy Plus Mini Kit (Qiagen, Germany). RNA quality and yield of each sample were assessed with the Qubit™ RNA BR Assay Kit (Thermo Fisher Scientific, USA). Samples were diluted to an average of 40 ng RNA/μl.

##### Sequencing and read processing

All samples were pooled, and one library (500 bp) was prepared for the Illumina sequencing (Illumina Truseq stranded mRNA). The library was paired-end sequenced with Illumina MiSeq v2 500 technology (read length 250 bp, intended output 7.5 Gbp, NucleomicsCore, Leuven). Read quality was tested using the software FastQC v0.11.2 [98]. Illumina PE reads that contained overrepresented Truseq LT or HT adaptor sequences were adapter-trimmed with Trimmomatic v0.35 [111], command line:

*TrimmomaticPE 0.32, ILLUMINACLIP: adaptors.fa:2:30:10 LEADING:3 TRAILING:3 MINLEN:36.*

then merged with BBMap v36.62 to produce 250 bp long single reads [112], command line:

*bbmerge-auto.sh rem k = 62 extend2 = 50 ecct vstrict.*

##### Transcriptome assembly

Merged reads were assembled to a transcriptome with SPAdes v3.10.1 ( [113], rnaspades.py -k 125). A K-mer size of 125 was chosen, since the merged reads were 250 bp long and the SPAdes developers advise using a K-mer length of half the read length for rnaSPAdes.

#### Structural and functional genome annotation

To support the gene model prediction, MiSeq PE 300 bp RNAseq reads were first mapped onto the genome assembly with STAR v2.5 [114], with poor results, since no annotation file was available yet to support the mapping. Mapping was thus repeated with Hisat2 [115]. The initial mapping with Hisat2 was better, but still poor due to reads spanning multiple short exons on typical *Artemia* genes with long introns, the 300 bp reads were cut back to multiple 50 bp PE reads to avoid single reads to span more than one intron, increasing mapping rates to 65%. From the resulting Hisat2 mapping with the shortened reads, intron border sequences were extracted from the genome to train SpliceMachine v1.2 using default parameters [116]. The obtained junctions (reads spanning an intron) were filtered on coverage (intron confirmed by at least 10 reads) and used as extrinsic information to support gene prediction. The software AUGUSTUS v3.3 [38] was trained (see Additional file 22: Augustus custom training files for *Artemia*) using the protein homology and RNA-seq provided as hints, to obtain a first draft annotation from which gene models were extracted and manually inspected to create a sufficiently large training set for EUGENE v4.2a [39]. After training, EuGene v4.2a was used with a custom parameter file for *Artemia* (see Additional file 23) and with the following information tracks: AUGUSTUS gene models, protein homology by BLAST+ v2.9.0 (SwissProt, Daphnia V4, *Artemia* proteins in NCBI, Refseq “invertebrate “ [117]), transcripts from non-inbred *Artemia* samples [34], and RNAseq from inbred *Artemia* as described above. Gene models were made available online for partners, for manual curation on the ORCAE platform [118] with GenomeView [119]. Genome assembly and annotation completeness was evaluated with the software BUSCO v3, using the Eukaryota dataset [120].

#### Identification of repeats

To explain the difference in genome size between branchiopods, the genomes analysed for comparative genomics were analysed for genome size, repeats, transposable elements, as well as exon and intron length and small RNAs as follows. As only for a subset of the investigated organisms, repeat data sets exist and as we aimed to have homogeneous repeat data to be able to compare repeats between species, we searched and collected repeats “de novo” in each branchiopod species mentioned in Table 1 (Methods section 2), including the *Artemia* genome, with RepeatModeler v1.0.8(−engine ncbi) [42]. The collected repeats were further annotated/described within the RepeatModeler v1.0.8 pipeline and subsequently combined with RepBase data [121] data for completeness and to identify cross-genome repeats.

#### Identification of mitochondrial and alien fragments

Scaffolds of mitochondrial origin were identified by BLAST+ v2.9.0 (blastp, Bit-score ≥ 50) of the *A. franciscana* mitochondrial genes [31] as indicated for homology searching [122] onto the *Artemia* proteome obtained by translation of the nuclear inbred genome assembly annotation. Scaffolds of equal or shorter length than the *A. franciscana* mitochondrial genome (15,822 bp), containing only mitochondrial genes were considered “mitochondrial” to retain only nuclear genome sequences.

Scaffolds originating from alien microbial, fungal or viral organisms were eliminated from the assembly after performing BLAST + v2.9.0 (blastx) on the RefSeq non-redundant protein databases “archaea”, “bacteria”, “protozoa”, “fungi”, “viral” and “invertebrate” [123], removing only scaffolds containing 100% non-invertebrate protein with a minimum ID of 70% and a maximum length of 5 kb without alien hits, to avoid eliminating scaffolds containing unknown arthropod genes.

#### Identification of functional genome content and repeats

Functional genome content and repeats were determined as described (Methods sections 1.5.2. and 1.5.3.) based on arthropod proteome databases (see Table 1, Methods section 2).

### Comparative genomics

Based on genome contig N50 (reflective of gap occurrence and thus potential gene completeness), and taxonomic group, 11 arthropod proteomes were selected (Table 1): one chelicerate as the outgroup (*Tetranychus urticae*), two insects with high-quality genome assemblies, one tardigrade as the representative extremophile, and 7 crustaceans including Amphipoda, Decapoda, Copepoda and Branchiopoda, thus including all four branchiopod lineages [124]. The first aim was to characterize unique properties of *Artemia* as compared to three other (non-extremophile) branchiopod species. The second aim was to find similarities between extremophilic tardigrade *H. dujardini* and *Artemia* on the one hand, and non-extremophile crustaceans on the other hand. The third aim was to find similarities between *Artemia* and *L. vannamei* pointing to biological processes in *L. vannamei* for which *Artemia* would be a suitable model.

For the *L. vannamei, H. azteca* and *Artemia* proteomes, only the longest isoform of each protein was used respectively. All proteomes were analysed with Orthofinder v2.3.5 [125]. Gene families were constructed and potential residual transposons still present in the predicted proteome were removed from the inbred genome annotation. A Venn diagram was constructed with shared groups of orthologs (orthogroups) between *Artemia* and branchiopod species with the software InteractiVenn [126].

Large and small gene family expansions were analysed between *Artemia* and other Branchiopoda, unique genes in *Artemia* and Tardigrada were compared to the other species in Table 1 and non-expanded or -contracted gene families were investigated between *Artemia* and *L. vannamei*. Orthologous groups containing at least 5 genes in *Artemia* with a number of genes N in species X were considered expanded or contracted respectively, compared to species Y, if N_x_ ≥ 3N_y_ or 3N_x_ ≤ N_y_. GO enrichment (Fisher’s exact test, FDR ≤ 0.05, OmicsBox) was performed onto expanded, contracted and common gene families to identify which biological processes were involved in 1) gene family expansion/contraction in *Artemia* compared to other Branchiopoda; 2) unique gene families in extremophiles *Artemia* and Tardigrada; 3) the non-expanded/contracted gene families in *Artemia* compared to *L. vannamei*. Enrichment was done with default settings: remove double ids was unchecked, rendering the Fisher’s Exact Test sensitive in the direction of the test: the sequences that are present in both test-set and reference set will be deleted from the reference, but not from the test set, which can lead to zero values in the reference set. For comparative genomics with Branchiopoda, results were reduced to the most specific GO’s (FDR < 0.05), and a bar chart with GO enrichment of up-and downregulated genes was made. For comparative genomics, with *L. vannamei*, a combined graph was made to show functional hierarchy of the non-expanded/contracted gene families in *Artemia* compared to *L. vannamei.*

### Functional genomics: salt and anoxia tolerance-specific genes

#### Sampling, RNA extraction, sequencing and read processing

All following experiments were subsequently done with *A. franciscana* of the strain San Francisco Bay (SFB), cyst bank ID code ARC1767. SFB was chosen and not GSL, because we were still working on the non-inbred (SFB x VC) genome at the time, and we had not yet sequenced the genome of the inbred *Artemia* (GSL).

##### Hydrated cysts

In order to synchronize the cyst metabolic stages, the following protocol was followed: cysts were hydrated in water, dehydrated in brine, rinsed and hydrated with tap water for 1 h with aeration, sieved and rinsed with mQ. Samples were taken with a small spatula cleaned with ethanol and put in labelled Eppendorfs, previously put in liquid N_2_ to flash-freeze. The remainder of the hydrated cysts was 1) put in filtered, non-autoclaved sea water brought to 30 g/L in a cone with aeration in the dark at 28 °C for 24 h to remove cysts hatching in the dark, the remaining cysts were left to hatch for 24 h under the same conditions, but in light, or 2) left to hydrate longer for a total of 6 h with aeration, and then used for testing under anoxic conditions.

##### Instar I/II nauplii, hatched in light, kept at low salinity

Nauplii were separated from cysts by removing aeration for a few min, pipetting the nauplii (25 ml pipette) over a sieve and adding them to a new, clean, aerated cone with Instant Ocean® USA 30 g/L.

Samples were taken after 15, 30, 45 and 60 min in new 30 g/L Instant Ocean® USA artificial sea water by transferring 50 ml (with 25 ml pipette) from a cone of nauplii over a sieve per sample time, rinsing with mQ over the sieve and taking 100 μl of mQ/*Artemia* mix per sample with a 1-ml pipette with cut tips. Samples were flash-frozen with liquid N_2_. Between sampling times, the sieve was washed with tap water and rinsed with autoclaved mQ.

To check the cysts/nauplii ratio in our samples (inevitably, some cysts or cyst shells cling to the nauplii) and the number of animals per sample, we observed 1 ml of the 50-ml samples and counted 93 instar I/II nauplii/ml and 9 cysts (full or empty)/ml, corresponding with 1550 nauplii per sample, each sample containing approximately 10% cysts (full or empty).

##### Instar I/II nauplii, hatched in light, kept at high salinity

Nauplii were separated from cysts by removing aeration for a few min, pipetting the nauplii (25-ml pipette) over a sieve and adding them to a new, clean, aerated cone with Instant Ocean® artificial brine at 200 g/L salinity.

Samples were taken after 15, 30, 45 and 60 min in new 200 g/L Instant Ocean® USA seawater by putting 50 ml (25 ml-pipette) from a cone of nauplii over a sieve per sample time, rinsing the sieve with mQ, and taking 100 μl of mQ/*Artemia* mix per sample with a 1 ml-pipette with cut tips. Samples were flash-frozen with liquid N_2_. Between sampling times, the sieve was washed with tap water and rinsed with autoclaved mQ.

##### Anoxic cysts

Two scintillation vials with cysts hydrated for 6 h in 30 g/L sea water at 28 °C were put under anoxic conditions in the light on a rotor. Anoxic conditions were reached according to a modified protocol [16]. In scintillation glass vials of 8 ml, 70–80 mg dry cysts were placed. N_2_ gas was flushed through the vial to remove the oxygen in the vial, then the vial was capped and set aside, allowing the N_2_ to seep into the porous parts of the outer cyst shell. N_2_ was bubbled through buffer (0.25 M NaCl in 0.05 M phosphate buffer, pH 7.2) during a minimum of 4 h. With the N_2_ still bubbling, 6 ml of the anoxic solution was transferred into each vial without any air leaking into the vial and Parafilm was wrapped around the tightly shut vial cap. Vials were oscillated on a rotating platform shaker (50 revolutions/min) for the first 24 h to hydrate the cysts completely and to ascertain that even traces of oxygen (if present) were consumed. The vials containing the anoxic, hydrated cysts were stored at room temperature and in ambient light.

Samples were taken after 25 h of anoxia (1 supplementary hour after metabolic activity stops). The content of each vial was sieved and rinsed with mQ. Per sample, 100 μl of mQ/*Artemia* mix was taken with a 1-ml pipette with cut tips. Samples were flash-frozen with liquid N_2_. Between sampling times, the sieve was washed with tap water and rinsed with autoclaved mQ.

The anoxic state in the scintillation vials was verified indirectly by observing the cysts under a binocular microscope after 3 days of anoxia. In a sample of 500 cysts, there was one clear umbrella and a few [2, 3] cysts possibly in the breaking stage. In similar conditions, but in the presence of oxygen, the same sample had a hatching percentage of 70% after 24 h. Crushing the cysts between microscope slides to push the non-hatched embryos out of their cyst shells was done to verify that practically all cysts contained an embryo and that the results were not biased by an excessive amount of empty cyst shells.

*A. franciscana* from the San Francisco Bay strain (SFB) were sampled under four different conditions, including different life cycle stages, and analysed (Table 2). RNA extraction of each sample, sequencing and read processing have previously been described [34]. Briefly, RNA was extracted from each freeze-dried, homogenized sample by a combined method with TRIzol (Invitrogen, USA), the RNeasy Mini Kit (Qiagen, Germany) and the DNase I Kit (Qiagen, Germany) [127]. RNA quality and yield of each sample were assessed with the Agilent 2100 bioanalyzer (Agilent Technologies, USA). Samples were diluted to an average of 40 ng RNA/μl. The RNA sample libraries were prepared by subsequent use of the TruSeq™ mRNA enrichment and TruSeq™ RNA sample preparation kits (Illumina, USA) and paired-end sequenced at the Genomics Core facility (UZ Leuven, http://gc.uzleuven.be) on an Illumina HiSeq 2000 instrument (2 × 100 bp, unstranded, insert size 200). Reads of each RNAseq library were quality-trimmed with CLC Assembly Cell v4.06 software [100].

#### Mapping and summarization

FastQC v 0.11.2 and MultiqQC, v1.7 [128] were used to evaluate the sequencing data quality [98, 128]. Two distinct GC content profiles were observed (Additional file 24: Sequence GC-content profiles for all samples used for differential expression analysis). Although both profiles are not problematic on their own, they clearly indicate a batch effect, possibly due to a separate library prep by the sequencing provider. As the batch effect was not associated with the contrasts of interest, yet constituted an unwanted source of variance, we took it into consideration during differential expression analysis as a random factor. Otherwise, no consistent infringements on data quality were identified. STAR v2.6.1c) was used to perform sequence alignment. To maximally preserve information, multi mapped reads were included in read summarization with the software featureCounts version1.5.2 [129].

#### Differential expression

The “high salinity” and “anoxia” conditions (Table 2) were examined for differential expression.

Samples were annotated based on life cycle stage, time point at which the sample was taken, salinity level of the treatment, light environment of the treatment and the batch effect, a technical variation source introduced by necessity in high-throughput studies (Table 5). Samples where a certain property was not tested are indicated as NA (non-applicable).

**Table 5:** Annotation of *A. franciscana* samples for differential expression analysis under different salinities and oxygen levels

Sample	Life cycle state	Time point (min)	Salinity	Light	Batch
HSR000198	cysts (anoxia)	60	NA	NA	1
HSR000210	cysts (anoxia)	60	NA	NA	1
HSR000222	cysts (anoxia)	60	NA	NA	1
HSR000190	nauplii	15	low	light	2
HSR000202	nauplii	15	low	light	1
HSR000214	nauplii	15	low	light	2
HSR000191	nauplii	30	low	light	2
HSR000203	nauplii	30	low	light	1
HSR000215	nauplii	30	low	light	2
HSR000192	nauplii	45	low	light	2
HSR000204	nauplii	45	low	light	1
HSR000216	nauplii	45	low	light	2
HSR000193	nauplii	60	low	light	2
HSR000205	nauplii	60	low	light	1
HSR000217	nauplii	60	low	light	2
HSR000194	nauplii	15	high	light	2
HSR000206	nauplii	15	high	light	1
HSR000218	nauplii	15	high	light	2
HSR000195	nauplii	30	high	light	2
HSR000207	nauplii	30	high	light	1
HSR000219	nauplii	30	high	light	2
HSR000196	nauplii	45	high	light	2
HSR000208	nauplii	45	high	light	1
HSR000220	nauplii	45	high	light	2
HSR000197	nauplii	60	high	light	2
HSR000209	nauplii	60	high	light	1
HSR000221	nauplii	60	high	light	2

##### High salinity and anoxia models

For high salinity, after filtering of non-informative genes, i.e., counts per million equivalent < 3 for over 14 out of 16 samples, the following model was fitted to the expression data (with the first batch, a low salinity treatment and the time point of 15 min as a reference):

$$ Y={\beta}_0+{\beta}_B{X}_B+{\beta}_S{X}_S+{\beta}_{T30}{X}_{T30}+{\beta}_{T45}{X}_{T45}+{\beta}_{T60}{X}_{T60} $$

$$ +{\beta}_{S:T30}{X}_S{X}_{T30}+{\beta}_{S:T45}{X}_S{X}_{T45}+{\beta}_{S:T60}{X}_S{X}_{T60} $$

With β_B_: batch effect of second batch.

β_S_: high salinity treatment effect.

β_T30_: time effect after 30 min (compared to 15 min).

β_T45_: time effect after 45 min (compared to 15 min).

β_T60_: time effect after 60 min (compared to 15 min).

This way, the effect of the salinity could be assessed at each time point separately. As the design is rather complex, a stage-wise testing approach as described by Van Den Berghe et al. was performed, thus boosting power for the interaction effects [130]. Briefly, an omnibus test was first performed to assess for which genes at least one of the contrasts of interest shows a significant difference (analogously to ANOVA). The contrasts of interest were the effect of salinity on each time point separately, and the average effect over all time points. Subsequently, a post-hoc analysis was performed for each contrast separately with an adjusted *p*-value.

For anoxia, after filtering of non-informative genes, i.e., counts per million < 3 for over 3 out of 6 samples, a simple model only considering the effect of anoxia compared to hydrated cysts was used. No batch effect was present in samples studied under anoxia.

Normalization was performed using trimmed mean of M-values (TMM), and FDR adjustment by Benjamini-Hochberg’s method, as default for edgeR v3.24.3, which was used for both omnibus test and post-hoc testing [131]. Genes with padjScreen < 0.05 (salinity) or PVal < 0.05 (anoxia) were considered differentially expressed and used for subsequent enrichment analyses.

#### Enrichment analysis

For each contrast, GO names (biological process, molecular process and cellular component), were analysed for enrichment (FDR ≤ 0.05) with Fisher’s exact test in OmicsBox [132] using the ID list of the selected DEG as the test set and the *Artemia* proteome (imported to OmicsBox, default InterProScan annotated, annotations merged) as the reference set. Enrichment was done with default settings: remove double ids was unchecked, rendering the Fisher’s Exact Test sensitive in the direction of the test: the sequences that are present in both test-set and reference set will be deleted from the reference, but not from the test set, which can lead to zero values in the reference set. Results were reduced to the most specific GO’s (FDR ≤ 0.05), and a bar chart with GO enrichment of differentially expressed genes was made. Pathway enrichment was performed with the online GUI software STRING v11.0 using default settings and model organism *Daphnia pulex* [133] and using “multiple sequences” as input: DEG under high salinity and a subset of DEG under anoxia containing only genes with a functional annotation (to conform to the 2 K sequence maximum of the platform) [133]. STRING first produced a “STRING annotation” by matching each protein set with *D. pulex* proteins in its database, only the best match was kept. Subsequently, STRING performed pathway enrichment with a stringent cut-off (FDR ≤ 0.05) on each *D. pulex* gene. Interesting enriched pathways were drawn with the online GUI software KEGG Mapper Search Pathway function [134] in the organism-specific mode (dpx).

## Supplementary Information

**Additional file 1.** Assembly characteristics of all assembled crustacean genomes. Characteristics listed are: species, whether the species genome is annotated yes or no, N50 of the fragments with the highest assembly hierarchy, number of fragments with the highest assembly hierarchy in the assembly, haploid genome size, assembly size, completeness of the assembly (=haploid GS/assembly size), taxonomic lineage (NCBI taxonomy), reference for the genome paper.

**Additional file 2.** Evolution of *Artemia* assembly quality metrics throughout the assembly steps. Evolution of the scaffold N50, the number of fragments and the genome completeness (assembly size/genome size) in the subsequent *Artemia* assembly stages

**Additional file 3 **BUSCO analysis results for the *A. franciscana* genome assembly and annotation.

**Additional file 4.** BLAST results for mitochondrial genes in the *Artemia* genome. Listed: Query accession and gene name, presence of a (significant) BLAST hit in the *Artemia* proteome with the highest bit score, E-value and bit score of the hit, scaffold length of the scaffold on which the hit lies, percentage of mitochondrial genes on this scaffold.

**Additional file 5.** Taxonomic groups of alien genomes identified in the *Artemia* genome.

**Additional file 6 **Expanded or contracted *Artemia* orthogroups compared to other Branchiopoda. Listed: Orthogroup ID, number of genes in this orthogroup in *A. franciscana*, *D. pulex*, *L. arcticus*, and *E. texana*, expanded or contracted status of the orthogroup in *Artemia* compared to *D. pulex*, *L. arcticus* and *E. texana*, conservation in Branchiopoda (whether this orthogroup contains genes for each branchiopod), comma-separated IPR description of *Artemia* genes in this orthogroup, *Artemia* genes in this orthogroup.

**Additional file 7 **GO enrichment of *Artemia* compared to other Branchiopoda. Listed: GO ID, name and category, false discovery rate (FDR) and *P* value of the Fisher’s exact test enrichment analysis in Blast2GO, number of *Artemia* genes from expanded/contracted orthogroups in this GO ID, number of whole *Artemia* genome genes in this GO category, number of *Artemia* genes from expanded/contracted orthogroups in this GO ID without GO annotation. The Fisher’s Exact Test is sensitive in the direction of the test: the genes that are present in the test-set and also in the reference genome set will be deleted from the reference, but not from the test set, resulting in zero sequences in the reference set and values above zero in the test set. Significantly enriched GOs (FDR ≤ 0.05, biological process) of *Artemia* genes in expanded or contracted orthogroups compared to Branchiopoda are given.

**Additional file 8 **Expanded or contracted *Artemia* and *H. dujardini* orthogroups compared to other Arthropoda. Listed: Orthogroup ID, number of genes in this orthogroup in *A. franciscana* and in the other arthropod species, expanded or contracted status of the orthogroup in *Artemia* compared to the other arthropod species, comma-separated IPR description of *Artemia* genes in this orthogroup, *H. dujardini* genes in this orthogroup, *Artemia* genes in this orthogroup.

**Additional file 9.** STAR mapping statistics for differential expression analysis in *Artemia*. Listed: sample name, total number of reads for this sample, percentage of uniquely mapped reads, absolute number of uniquely mapped reads, percentage of multi mapped reads, absolute number of multi mapped reads.

**Additional file 10.** Summarization statistics for differential expression analysis in *Artemia*. Listed: sample name, total counts, percentage of counts assigned to a gene annotation, absolute counts assigned to a gene annotation. * notice that this amount can be more than the sum of uniquely mapped + multi-mapped in the mapping statistics since multi-mapped reads are considered.

**Additional file 11 **Differentially expressed genes under high salinity (*p* < 0.05). Listed: functional annotation of the differentially expressed gene, gene ID in the genome annotation and on the ORCAE platform, *p* value, average log fold change of gene expression under high salinity, gene regulation of the differentially expressed gene (up or down), InterPro description of the gene family to which the gene belongs.

**Additional file 12 **Differentially expressed genes under anoxia (*p* < 0.05). Listed: functional annotation of the differentially expressed gene, gene ID in the genome annotation and on the ORCAE platform, p value, log fold change of gene expression under anoxia, gene regulation of the differentially expressed gene (up or down), InterPro description of the gene family to which the gene belongs.

**Additional file 13 **GO enrichment in *Artemia* under high salinity. Significantly Enriched GOs (FDR ≤ 0.05) of *Artemia* genes differentially expressed under high salinity. Listed: GO ID, name and category, false discovery rate (FDR) and *P* value of the Fisher’s exact test enrichment analysis in Blast2GO, number of DEG under high salinity in this GO category, number of whole *Artemia* genome genes in this GO category, number of DEG under high salinity without GO annotation. The Fisher’s Exact Test is sensitive in the direction of the test: the genes that are present in the test-set and also in the reference genome set will be deleted from the reference, but not from the test set, resulting in zero sequences in the reference set and values above zero in the test set.

**Additional file 14 **Pathway enrichment in *Artemia* under high salinity. Significantly enriched (Fisher’s exact test corrected for multiple testing, FDR ≤ 0.05) pathways of *Artemia* genes differentially expressed under high salinity. Listed in first worksheet (STRING annotation): gene number, ORCAE gene ID, STRING *Daphnia pulex* gene ID, BLAST identity and bit score, gene name and gene annotation. Listed in second worksheet (STRING pathway enrichment): KEGG *Daphnia pulex* pathway name, pathway description, number of DEG under high salinity in this pathway, number of genes in the *D. pulex* genome that belong to this pathway, enrichment FDR, matching *D. pulex* gene IDs, matching gene names in pathways shown in figures and additional files, matching *D. pulex* gene ID labels.

**Additional file 15.** Consolidation of DEG analysis, GO enrichment and pathway enrichment in *Artemia* under high salinity.

**Additional file 16.** The enriched Carbon metabolism pathway in *Artemia* under high salinity. Up- and downregulated genes are indicated on the KEGG map dpx01200.

**Additional file 17.** GO enrichment in *Artemia* under anoxia. Significantly enriched GOs (FDR ≤ 0.05) of *Artemia* genes differentially expressed under anoxia. Listed: GO ID, name and category, false discovery rate (FDR) and P value of the Fisher’s exact test enrichment analysis in Blast2GO, number of DEG under anoxia in this GO ID, number of whole *Artemia* genome genes in this GO ID, number of DEG under anoxia without GO annotation. The Fisher’s Exact Test is sensitive in the direction of the test: the genes that are present in the test set and also in the reference genome set will be deleted from the reference, but not from the test set, resulting in zero sequences in the reference set and values above zero in the test set.

**Additional file 18 **Pathway enrichment in *Artemia* under anoxia. Significantly enriched (Fisher’s exact test corrected for multiple testing, FDR ≤ 0.05) pathways of *Artemia* genes differentially expressed under anoxia. Listed in first worksheet (STRING annotation): gene number, ORCAE gene ID, STRING *Daphnia pulex* gene ID, BLAST identity and bit score, gene name and gene annotation. Listed in second worksheet (STRING pathway enrichment): KEGG *Daphnia pulex* pathway name, pathway description, number of DEG under anoxia in this pathway, number of genes in the *D. pulex* genome that belong to this pathway, enrichment FDR, matching *D. pulex* gene IDs, matching gene names in pathways shown in figures and additional files, matching *D. pulex* gene ID labels.

**Additional file 19.** Consolidation of DEG analysis, GO enrichment and pathway enrichment in *Artemia* under anoxia.

**Additional file 20.** The enriched N-glycan biosynthesis pathway in *Artemia* under anoxia. Up- and downregulated genes are indicated on the KEGG map dpx00510.

**Additional file 21.** The enriched Basal transcription factors pathway in *Artemia* under anoxia. Up- and downregulated genes are indicated on the KEGG map dpx03022.

**Additional file 22.** Augustus custom training files for *Artemia*. Includes probabilities, parameters and weights used for Augustus training for annotation of the *Artemia* genome.

**Additional file 23.** EuGene custom parameter file for *Artemia*. Includes parameters used for EuGene training for annotation of the *Artemia* genome.

**Additional file 24.** Sequence GC-content profiles for all samples used for differential expression analysis.

## Data Availability

The datasets supporting the conclusions of this article are available in the GENBANK repository, Inbred *A. franciscana* genome: SUB6538872. Bioproject PRJNA589114. Inbred *A. franciscana* transcriptome: Bioproject PRJNA589261. This submission is deposited in Genbank under the code “JAGUUB000000000” and will be made public after acceptation of this paper. For reviewers of this paper, a confidential account was made on our genome platform Orcae: https://bioinformatics.psb.ugent.be/orcae/ login: artemia. password: AuBSvP. The sequencing read datasets used and/or analysed during the current study are available from the corresponding author on reasonable request.
