# Supplementary figures and images for: The genome of the extremophile Artemia provides insight into strategies to cope with extreme environments
Source: BMC Genomics. 2021 Aug 31;22:635. doi: 10.1186/s12864-021-07937-z (PMC8406910; doi:10.1186/s12864-021-07937-z)

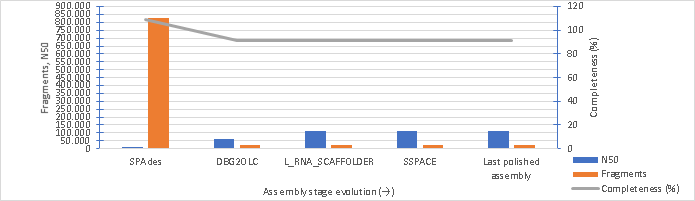

Supplement: Supplementary file 2 — Additional file 2. Evolution of Artemia assembly quality metrics throughout the assembly steps. Evolution of the scaffold N50, the number of fragments and the genome completeness (assembly size/genome size) in the subsequent Artemia assembly stages [file 12864_2021_7937_MOESM2_ESM.png]

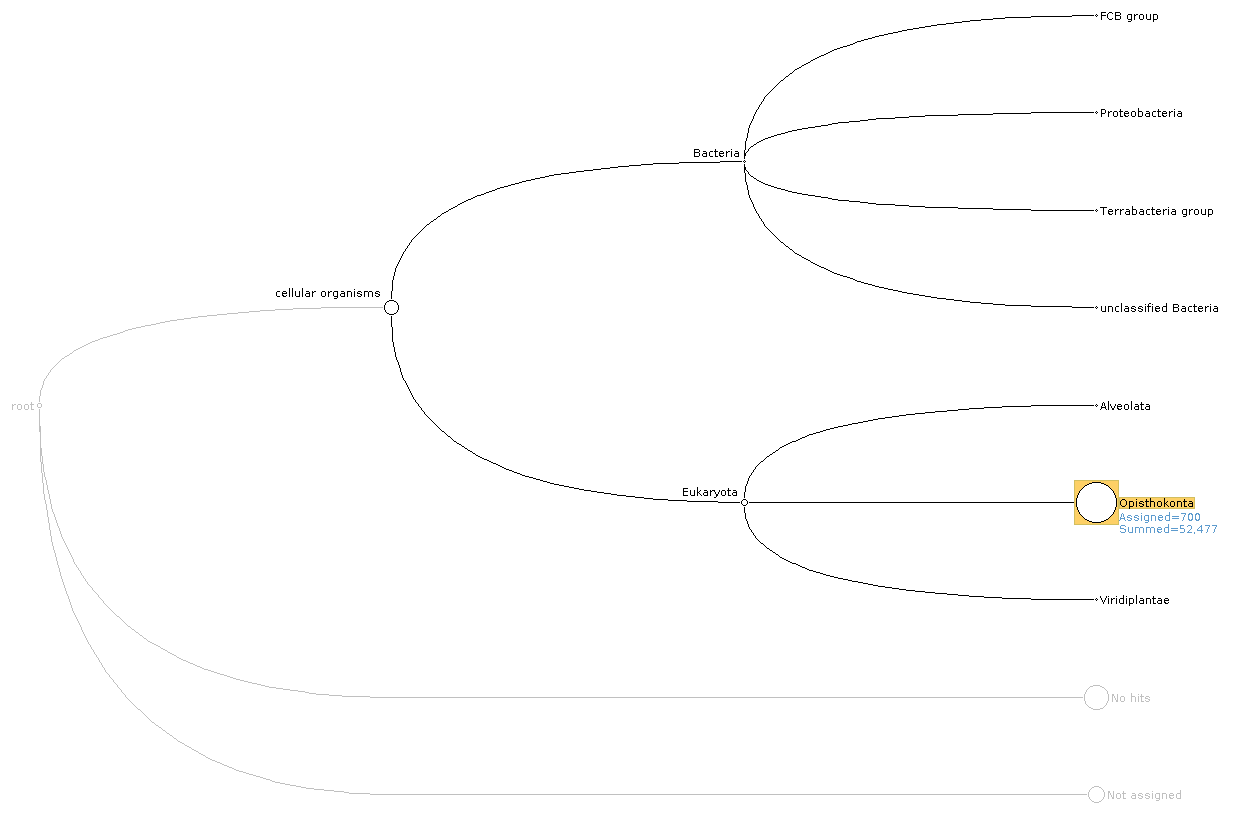

Supplement: Supplementary file 5 — Additional file 5. Taxonomic groups of alien genomes identified in the Artemia genome. [file 12864_2021_7937_MOESM5_ESM.png]

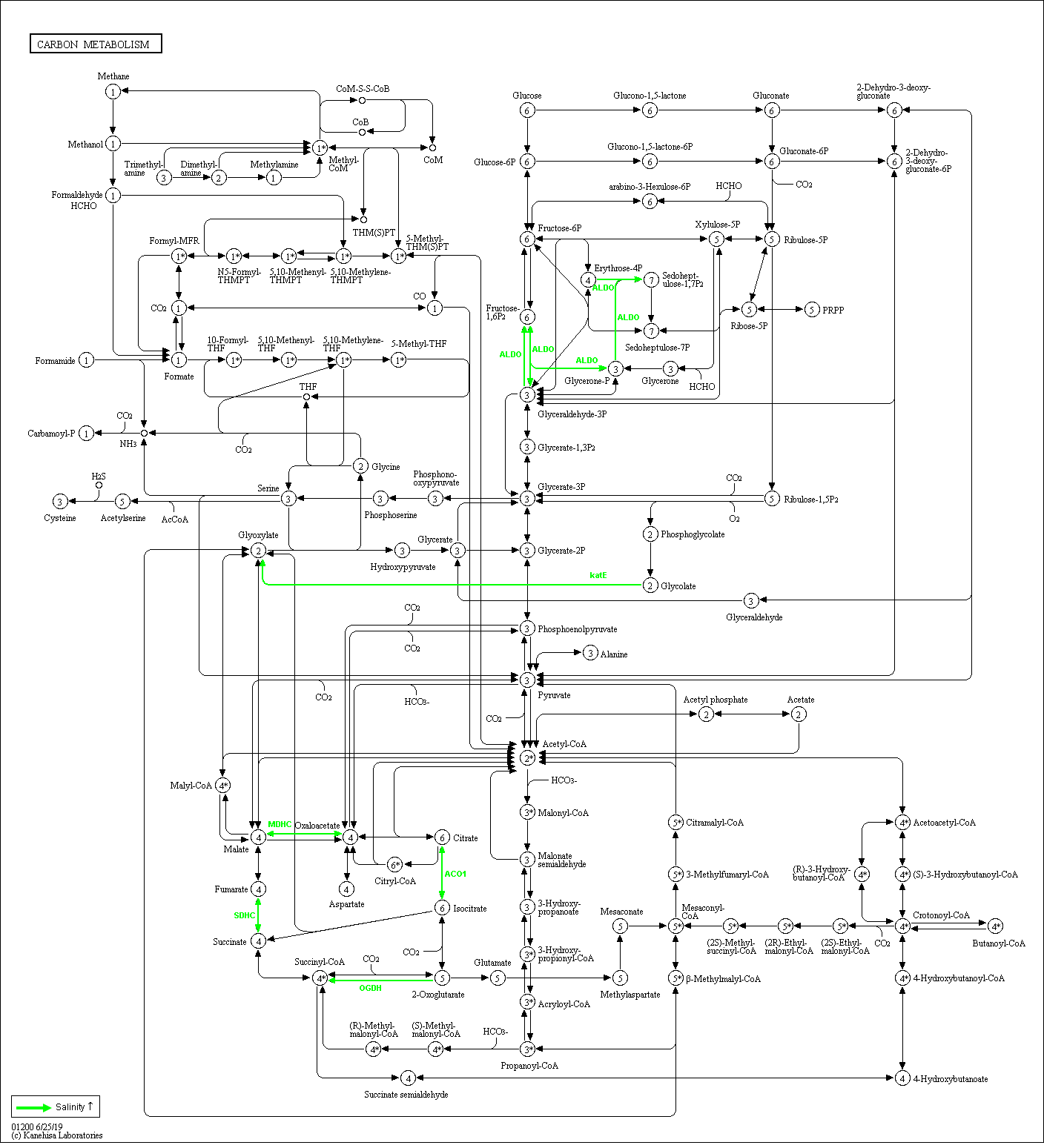

Supplement: Supplementary file 16 — Additional file 16. The enriched Carbon metabolism pathway in Artemia under high salinity. Up- and downregulated genes are indicated on the KEGG map dpx01200. [file 12864_2021_7937_MOESM16_ESM.png]

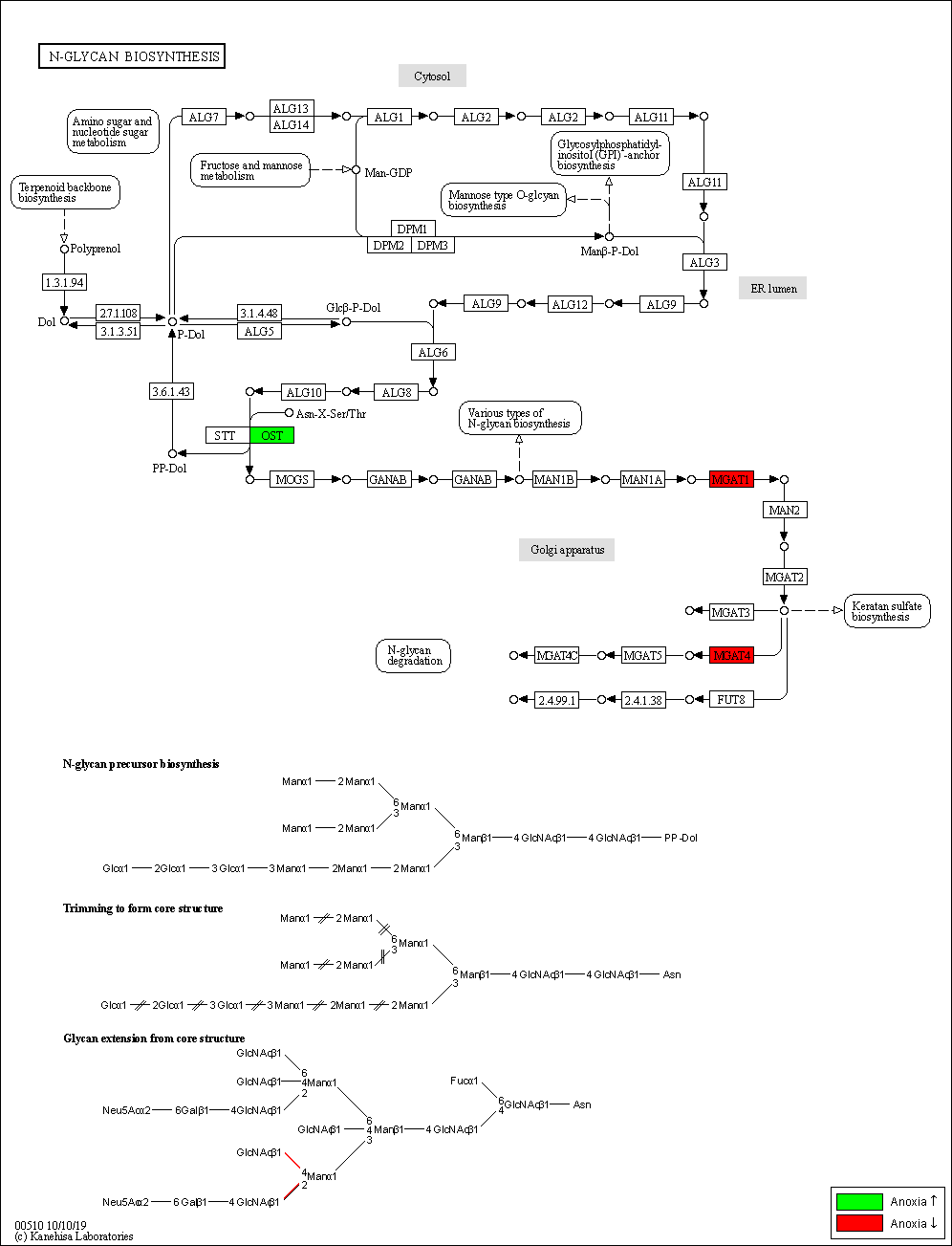

Supplement: Supplementary file 20 — Additional file 20. The enriched N-glycan biosynthesis pathway in Artemia under anoxia. Up- and downregulated genes are indicated on the KEGG map dpx00510. [file 12864_2021_7937_MOESM20_ESM.png]

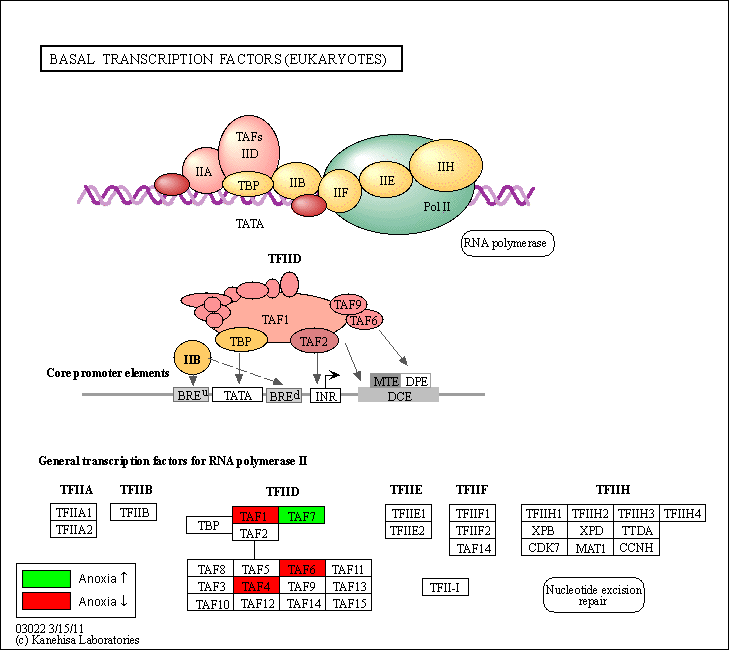

Supplement: Supplementary file 21 — Additional file 21. The enriched Basal transcription factors pathway in Artemia under anoxia. Up- and downregulated genes are indicated on the KEGG map dpx03022. [file 12864_2021_7937_MOESM21_ESM.png]

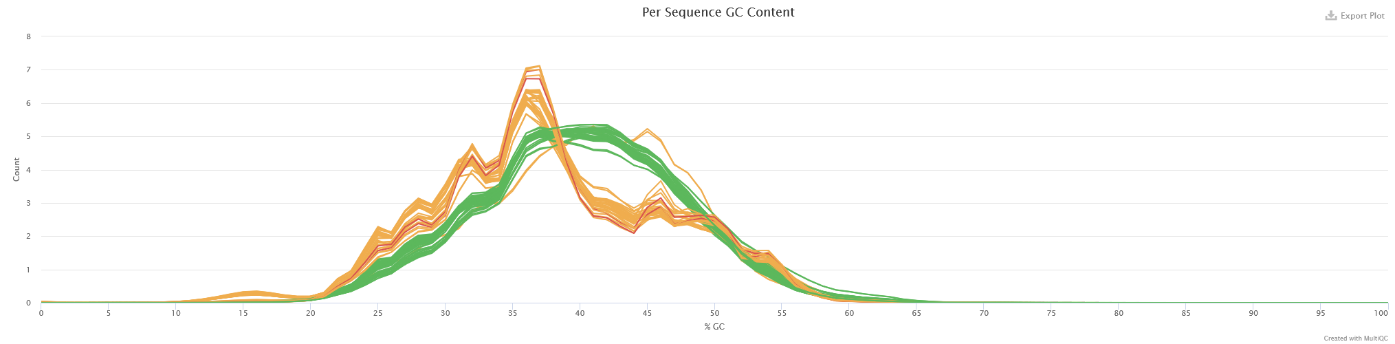

Supplement: Supplementary file 24 — Additional file 24. Sequence GC-content profiles for all samples used for differential expression analysis. [file 12864_2021_7937_MOESM24_ESM.png]
